# Subcutaneous fat necrosis in newborns: a systematic literature review of case reports and model of pathophysiology

**DOI:** 10.1186/s40348-022-00151-1

**Published:** 2022-11-24

**Authors:** Leonie Frank, Stephanie Brandt, Martin Wabitsch

**Affiliations:** 1 Division of Pediatric Endocrinology and Diabetes, Department of Pediatrics and Adolescent Medicine, University Medical Center Ulm, Ulm, Germany; 2 Department of Orthopaedics and Trauma Surgery, Oberschwaben Clinic Wangen im Allgäu, Wangen im Allgäu, Germany

**Keywords:** Subcutaneous fat necrosis of the newborn, SCFN, Children, Newborn, Hypercalcaemia

## Abstract

**Background:**

Subcutaneous fat necrosis of the newborn (SCFN) is a rare disease occurring in the first days of life. Characteristically, the infants show hard nodules in subcutaneous tissue, purple or erythematous in color and appear on the upper back, cheeks, buttocks and limbs. In most cases, SCFN is a self-limiting disease, as the nodules disappear in up to 6 months. A severe complication associated with SCFN is hypercalcaemia. Pathophysiological mechanisms causing SCFN or associated hypercalcaemia are not fully understood yet.

**Methods:**

A systematic literature research including the six biggest databases for medical research has been used to identify all published case reports of SCFN. *N* = 206 publications has been identified containing *n* = 320 case reports. All cases have been classified into four subgroups (depending on reported serum-calcium-level): hypercalcaemia, normocalcaemia, hypocalcaemia or no information given. Reported maternal factors, birth characteristics, details about SCFN, diagnostics, therapy and long-term observations have been extracted from publications.

**Results:**

This is the first systematic literature research that summed up all published cases of SCFN from 1948 up to 2018. Information about serum calcium level was given in 64.3% of the cases. From those, the majority showed hypercalcaemia (70.5%) (normocalcaemia 25.1%, hypocalcemia 4.3%). 89.3% of newborns with hypercalcaemia showed suppressed levels of the parathormone. Maternal gestational diabetes, maternal hypertensive diseases during pregnancy, macrosomia (> 4000g), asphyxia and therapeutic hypothermia are risk factors for SCFN. Histological findings showed a granulomatous inflammation in 98% of cases.

**Conclusion:**

We identified that maternal, birth characteristics and therapeutic measures are probably risk factors for SCFN. These risk factors should be taken into account within the care of neonates.

**Supplementary Information:**

The online version contains supplementary material available at 10.1186/s40348-022-00151-1.

## Background

Subcutaneous fat necrosis (SCFN) is a very rare form of panniculitis in the newborn, in which inflammation of the subcutaneous adipose tissue usually occurs in the first days after birth [1]. It usually affects mature or transferred infants, who clinically present with single or multiple erythematous nodular/plaque-like indurations. The back, shoulders, buttocks, cheeks and extremities are considered predisposed sites. In addition to maternal factors such as gestational diabetes or maternal hypertension, birth asphyxia, meconium aspiration, or therapeutic hypothermia for the treatment of perinatally occurring hypoxic-ischemic encephalopathies (HIE) are thought to be risk factors for the development of subcutaneous adipose tissue necrosis. In most cases, the disease has a good prognosis with complete healing within several weeks to months. However, various complications can occur, which can sometimes have long-term consequences in children with SCFN. Long-term complications of neonatal subcutaneous fat necrosis include atrophic scars at the affected skin sites and persistent nephrocalcinosis. The most important acute complication of SCFN is hypercalcaemia, which should be diagnosed and treated in time because of its possible lethal course. Clinical indications for this are vomiting, somnolence, muscle weakness, nephrocalcinosis and cardiac arrhythmias.

The underlying pathophysiological of SCFN is not yet sufficiently understood. Several possible causes and mechanisms are discussed in the literature: (a) less perfusion leads to hypoxic cellular damage of the subcutaneous adipose tissue [2], (b) the special composition of neonatal adipose tissue causes crystallisation of the fat during hypothermia [3], (c) mechanical pressure is a triggering factor of tissue hypoperfusion [4], (d) there is a relationship between hypothermia/birth complications and the function or localisation of brown adipose tissue [5]. Concomitant hypercalcaemia is one of the most common complications of subcutaneous adipose tissue necrosis in the newborn. This is thought to be caused by the following mechanisms: extrarenal vitamin D secretion by macrophages [6, 7] and direct calcium release from the skin lesions [8–10]. Holzel first suggested a relationship between the occurrence of asphyctic events during birth and the development of subcutaneous adipose tissue necrosis [2]. In perinatal asphyxia, hypoxia and hypercapnia occur and are triggered by impaired placental or pulmonary gas exchange in the neonate [11]. The hypoxia that occurs leads to the initiation of the diving reflex in the neonate as well as increased activity of the sympathetic nervous system [12, 13]. As a result, there is a shift in blood volume towards vital organs such as the heart, brain, and adrenal glands and concomitant generalised peripheral vasoconstriction [13, 14]. As a result, the perfusion of cutaneous tissues also decreases. Another cause that leads to a decrease in perfusion in the cutaneous tissue due to vasoconstriction is a drop in temperature as in the case of therapeutic hypothermia [15–17].

The aim of the systematic review was to summarise previously published case reports of neonates with subcutaneous fat necrosis and to develop a pathophysiological model for the SCFN on the basis of findings from literature review.

## Methods

### Literature research

Six literature databases (PubMed/MEDLINE, OvidSP, Cochrane Library, BIOSIS, DIMDI, Google Scholar) were searched for defined search terms. The search terms listed below were used using Boolean operators as part of the systematic literature search: subcutaneous fat necrosis AND newborn, subcutaneous fat necrosis AND hypercalcaemia, fat necrosis AND newborn AND hypercalcaemia, fat necrosis AND newborn AND case, fat necrosis AND newborn AND case, subcutaneous fat necrosis AND nephrocalcinosis, subcutaneous fat necrosis AND vitamin D, subcutaneous fat necrosis AND hypocalcaemia, “subcutaneous fat necrosis” AND “case report”. EndNote 20 (Alfasoft GmbH, Frankfurt, Germany) was used as reference management tool. The first step consisted of examining all titles or abstracts according to defined inclusion and exclusion criteria. Inclusion criteria were defined as: Confirmed SCFN diagnosis, case report or case series, newborn/child < 2 years and publication date between 1949 and 2018. Exclusion criteria were defined as: No case report or case series, other underlying disease (e.g. pancreatitis), report from animals, animal experiments, age of the patient > 2 years, text or abstract not available in English, German, Spanish or Italian. The remaining texts were included in the second step. This consisted of screening the full texts according to inclusion and exclusion criteria. The following mentioned parameters were collected in a database per case report/case series: (1) maternal factors: age at birth of the child (years), gravida (number), maternal risk factors (e.g. previous diseases), risk factors during pregnancy (e.g. maternal smoking), (2) birth characteristics: sex, gestational week, mode of birth (vaginal or sectio ceasarea), birth weight (grams), APGAR score, birth complications and their therapy, diagnostics in newborn shortly after birth, (3) characteristics of the SCFN: age at appearance of typical skin lesions, time point and age at diagnosis, type of diagnosis (clinical or histological), age at onset of electrolyte disturbance, macroscopic and microscopic description of skin lesions, additional symptoms, diagnostic (laboratory findings, further diagnostics e.g. sonography, electrocardiogram, therapy (administered drugs (time, amount, duration)), (4) course of the disease: time point of healing, time point of the last follow-up, duration between appearance of subcutaneous fat necrosis and electrolyte disturbance or between appearance and healing of skin lesions, long-term consequences.

### Definition of sub-groups

To identify possible differences between patients with or without concomitant hypercalcaemia, the patient population was divided into four subgroups: (a) patients with laboratory-proven hypercalcaemia (*n* = 146), (b) patients with laboratory-proven normocalcaemia (*n* = 52), (c) patients with laboratory-proven hypocalcaemia (*n* = 9) and (d) patients without laboratory information regarding calcium levels (*n* = 113).

## Results

### Literature research

**Fig. 1:**
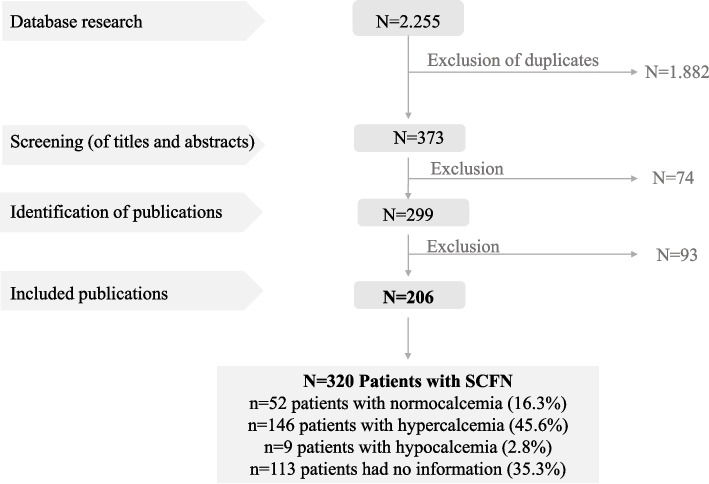
Flow chart of systematic literature research

The systematic literature search resulted in a total number of *n* = 2.255 articles (Fig. 1). Due to the search terms used, duplicates occurred both within the individual databases searched and between the various databases themselves. These duplicates were identified and automatically removed using the literature management programme. A number of *n* = 373 articles remained, which corresponded to the pre-selection. In the selection process that followed the systematic literature search, *n* = 74 articles were excluded in the first screening. *N* = 299 articles were included in the full-text analysis (Table S1). In the second selection step, a further *n* = 93 articles were excluded with indication of the respective reason (Table S2). A total of *n* = 206 publications were finally included in the analysis. Because some publications described multiple case reports, this resulted in a collective of *n* = 320 reported cases with diagnosed SCFN. Distribution of patients among subgroups revealed *n* = 52 patients with normocalcaemia (16.3%), *n* = 146 patients with hypercalcaemia (45.6%), *n* = 9 patients with hypocalcaemia (2.8%) and *n* = 113 patients had no information in this regard (35.3%).

### Clinical characteristics of subcutaneous fat necrosis (SCFN)

#### Diagnosis

In 34.1% (*n* = 109) of all cases, the diagnosis of subcutaneous fat necrosis was made on the basis of the clinical symptoms. In another 50% (*n* = 160) of the case reports, histological examination was indicated in case of existing clinical suspicion, which contributed to the diagnosis. Lower proportions were diagnosed by radiological methods (*n* = 17, 5.3%) and postmortem diagnosis (*n* = 4, 1.3%). In *n* = 30, no indication of the type of diagnosis was given (data not shown).

#### Time of diagnosis

The diagnosis of subcutaneous fat necrosis (SCFN) in newborns occurred in a short time interval after delivery (median 7 days) (Table 1). In the group of patients with hypercalcaemia, the diagnosis took place 1 day earlier (median 6.5 days) than in the group of patients with normocalcaemia (median 7.5 days). Exclusively in the group of patients with hypercalcaemia, subcutaneous adipose tissue necrosis also occurred at ≥ 30 days of age. In 3.4% of all cases (*n* = 11), the occurrence of subcutaneous fat necrosis was diagnosed in temporal connection with a performed surgery. The time interval between the operation and the appearance of the skin lesions was 14–35 days. Among the *n* = 11 cases, *n* = 10 operations on the heart were described, as well as *n* = 1 operation of an abdominal hernia. In *n* = 5 patients, hypothermia treatment was performed during surgery.

**Table 1:** Characteristics of SCFN in newborns

			Subgroups			
		All patients (*n* = 320)	Normocalcaemia (*n* = 52)	Hypercalcaemia (*n* = 146)	Hypocalcaemia (*n* = 9)	No information about calcium level (*n* = 113)
Age at diagnosis of SCFN [days after birth]	*N*	297	42	136	8	108
	mean ± SD	11.3 ± 11.6	11.1 ± 9.1	11.8 ± 12.0	6.3 ± 5.8	11.4 ± 12.3
Size of skin lesion (cm^2^)	*N*	83	20	36	1	26
	mean ± SD	17.3 ± 38.1	19.0 ± 33.8	24.3 ± 50.3	3.8 ± 0	8.1 ± 9.8
Symptoms at time of diagnosis	*N*	285	22	194	6	63
Radial paresis	*N* (%)	30 (10.5)	1 (4.5)	0 (0.0)	0 (0.0)	29 (46.0)
Vomiting, diarrhoea	*N* (%)	29 (10.2)	1 (4.5)	25 (12.9)	0 (0.0)	3 (4.8)
Failure to thrive	*N* (%)	28 (9.8)	1 (4.5)	27 (13.9)	0 (0.0)	0 (0.0)
Fever	*N* (%)	25 (8.8)	3 (13.6)	17 (8.7)	0 (0.0)	5 (7.9)
Heart disease	*N* (%)	21 (7.4)	6 (27.3)	13 (6.7)	2 (33.3)	0 (0.0)
Weight loss	*N* (%)	18 (6.3)	1 (4.5)	16 (8.2)	0 (0.0)	1 (1.6)
Poor feeding (breast milk/formula)	*N* (%)	18 (6.3)	2 (9)	15 (7.7)	0 (0.0)	1 (1.6)
Increased excitability	*N* (%)	17 (6.0)	0 (0.0)	16 (8.2)	0 (0.0)	1 (1.6)
Muscular hypotonia	*N* (%)	11 (3.9)	0 (0.0)	8 (4.1)	1 (16.7)	2 (3.2)
Lethargy	*N* (%)	11 (3.9)	0 (0.0)	8 (4.1)	0 (0.0)	3 (4.8)
Dehydration	*N* (%)	10 (3.5)	0 (0.0)	8 (4.1)	0 (0.0)	3 (4.8)
Further^a^	*N* (%)	37 (23.5)	6 (27.3)	41 (21.1)	3 (50.0)	17 (27.0)
Serum calcium concentration [mmol/l]	*N*	–	–	111	–	–
	Mean ± SD	–	–	3.6 ± 0.6	–	–
Ionised calcium [mmol/l]	*N*	–	–	19	–	–
	Mean ± SD	–	–	1.8 ± 1.0	–	–
Vitamin D concentration (25(oH)D3) [ng/ml]	*N*	33	–	–	–	–
	Mean ± SD	23.9 ± 15.8	–	–	–	–
1,25(OH)2D3 [pmol/l]	*N*	39	–	–	–	–
	Mean ± SD	212.9 ± 135.3	–	–	–	–
Parathormon concentration [pmol/l]	*N*	28	–	–	–	–
	Mean ± SD	14.1 ± 38.5	–	–	–	–
Serum triglycerid concentration [mg/dl]	*N*	18	2	14	2	–
	Mean ± SD	346.3 ± 177.9	214 ± 75	383.3 ± 183.5	219.2 ± 12.7	–
Time between diagnosis of SCFN and electrolyte disturbance	*N*	122	–	114	8	–
	Mean ± SD	18.6 ± 32.2	–	19.3 ± 32.7	8.8 ± 24.0	–
Calcifications in relation to localisation	*N*	97	5	85	1	6
Kidney	*N* (%)	46 (47.4)	0 (0.0)	45 (53.0)	0 (0.0)	1 (16.7)
subcutanous	*N* (%)	36 (37.1)	5 (100)	26 (30.6)	1 (100)	4 (66.7)
Brain	*N* (%)	4 (4.1)	0 (0.0)	4 (4.7)	0 (0.0)	0 (0.0)
Myocard	*N* (%)	3 (3.1)	0 (0.0)	2 (2.4)	0 (0.0)	1 (16.7)
Liver	*N* (%)	3 (3.1)	0 (0.0)	3 (3.5)	0 (0.0)	0 (0.0)
Blood vessels	*N* (%)	2 (2.1)	0 (0.0)	2 (2.4)	0 (0.0)	0 (0.0)
Stomach	*N* (%)	1 (1.0)	0 (0.0)	1 (1.2)	0 (0.0)	0 (0.0)
Bronchia	*N* (%)	1 (1.0)	0 (0.0)	1 (1.2)	0 (0.0)	0 (0.0)
Metaphyses	*N* (%)	1 (1.0)	0 (0.0)	1 (1.2)	0 (0.0)	0 (0.0)
Symptoms and course of SCFN	*N*	140	26	65	8	41
duration until healing [days]	Mean ± SD	90.8 ± 65.1	90.8 ± 60.2	100.6 ± 78.3	79.5 ± 43.9	77.3 ± 41.8

#### Localisation

**Fig. 2:**
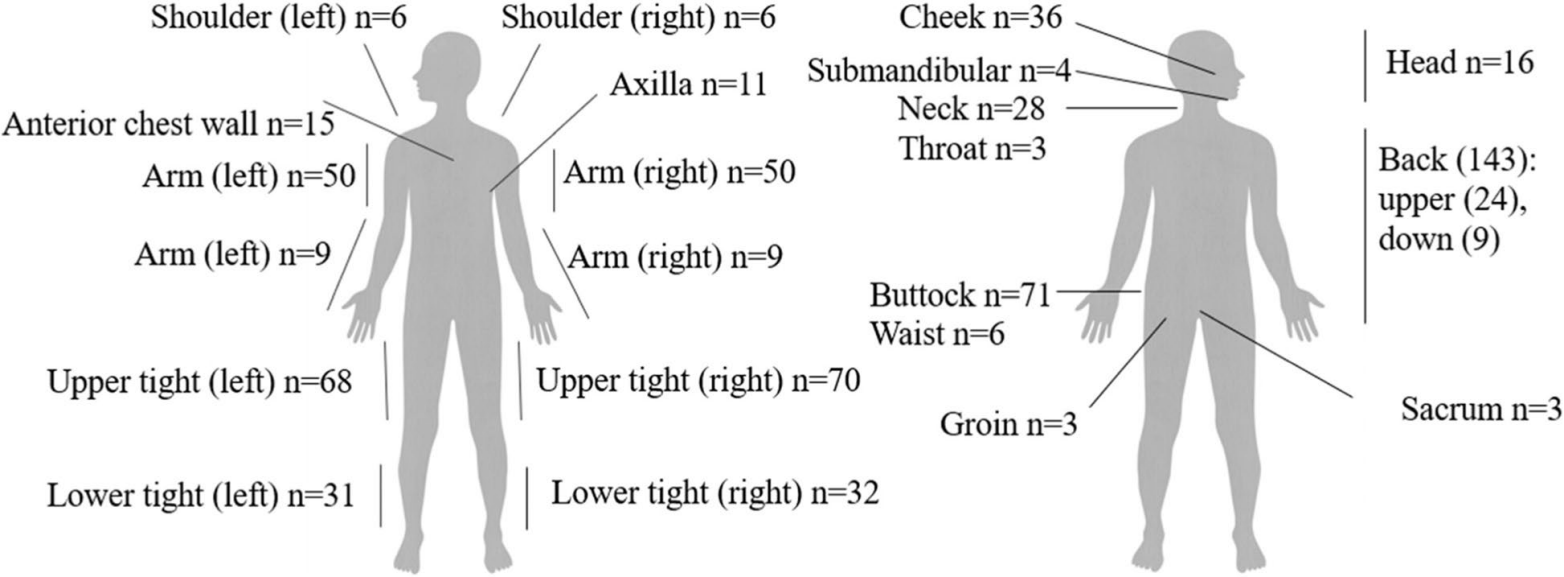
Reported localisation of subcutaneous fat necrosis in patients (*n* =number of patients in whom it was reported)

The characteristic skin lesions were reported in *n* = 176 children (85.4%) and were most frequently located on the back (21.7%), followed by the thighs (17%), upper arms (14.2%) and buttocks (9.3%) (Fig. 2). In *n* = 320 case reports, *n* = 812 skin lesions were described. In each child, the characteristic necrosis occurred in three different localisations (data not shown).

#### Size of necrosis

The size of the necrosis varied greatly from 0.1 × 0.4 cm to 15 × 19 cm (Table 1). A tendency towards larger skin lesions was observed in the group of newborns with hypercalcaemia compared to the other two groups of newborns dependent on serum calcium concentration (normo- and hypocalcaemia). Size of the lesion was weakly positively correlated with serum calcium concentration (*r* = 0.23; *p* > 0.05, data not shown).

#### Pathological finding of lesion

A description of the histologic picture was available for *n* = 157 case reports. In patients in whom histological examination was performed, characteristic features of SCFN were described, including necrosis of adipocytes in the subcutaneous tissue with surrounding granulomatous reaction, an inflammatory infiltrate of lymphocytes, neutrophilic granulocytes, histiocytes and multinuclear giant cells in the subcutis, and partial deposition of calcium salts in the necrotic tissue (data not shown).

### Clinical characteristics of neonates with subcutaneous fat necrosis (SCFN)

#### Gender distribution

Males and females were equally affected (*n* = 149 males vs. *n* = 158 females) (Table 2). In the group of neonates with hypocalcaemia (*n* = 8), primarily females were affected (*n* = 7 females versus *n* = 1 male).

**Table 2:** Birth characteristics in newborns with SCFN

			subgroups			
		All patients (*n* = 320)	Normocalcaemia (*n* = 52)	Hypercalcaemia (*n* = 146)	Hypocalcaemia (*n* = 9)	No information about calcium level (*n* = 113)
Sex	*N*	307	50	138	8	111
Male	*N* (%)	149 (48.5)	24 (48)	65 (47.1)	1 (12.5)	59 (53.1)
Female	*N* (%)	158 (51.5)	26 (52)	73 (52.9)	7 (87.5)	52 (46.9)
Birth weight	*N*	218	35	91	4	88
	Mean ± SD	3606±759	3232±937	3873±650	2975±763	3638±560
APGAR score ≤ 3	*N*	115	19	68	3	15
1 min	*N* (%)	78 (61.4)	12 (46.2)	47 (68.1)	3 (66)	9 (59.3)
Complications that occurred during or in the first days after birth	*N*	522	78	284	17	146
Asphyxia	*N* (%)	62 (11.9)	8 (10.3)	30 (10.6)	4 (23.5)	20 (13.7)
Respiratory distress	*N* (%)	49 (9.4)	10 (12.8)	26 (9.2)	2 (11.8)	11 (7.5)
Meconium aspiration	*N* (%)	48 (9.2)	3 (3.8)	35 (12.3)	0 (0.0)	10 (6.8)
Meconium in amniotic fluid	*N* (%)	41 (7.9)	6 (7.7)	18 (6.3)	3 (17.6)	14 (9.6)
Hypoglycaemia	*N* (%)	41 (7.9)	4 (5.1)	22 (7.7)	1 (5.9)	15 (10.3)
Hypoxic-ischemic encephalopathy	*N* (%)	35 (6.7)	8 (10.3)	21 (7.4)	1 (5.9)	5 (3.4)
Cerebral seizures	*N* (%)	27 (5.2)	4 (5.1)	18 (6.3)	0 (0.0)	5 (3.4)
Sepsis or infection	*N* (%)	22 (4.2)	0 (0.0)	11 (3.9)	0 (0.0)	11 (7.5)
Hypoxaemia	*N* (%)	20 (3.8)	0 (0.0)	10 (3.5)	0 (0.0)	8 (5.5)
Peripheral cyanosis	*N* (%)	20 (3.8)	5 (6.4)	7 (2.5)	0 (0.0)	8 (5.5)
Tachypnea	*N* (%)	14 (2.7)	2 (2.6)	5 (1.8)	2 (11.8)	5 (3.4)
Hypotension	*N* (%)	14 (2.7)	2 (2.6)	9 (3.2)	1 (5.9)	2 (1.7)
Anaemia/thrombocytopenia	*N* (%)	14 (2.7)	2 (2.6)	5 (1.8)	0 (0.0)	8 (5.8)
Acute renal failure	*N* (%)	10 (2.0)	2 (2.6)	7 (2.5)	0 (0.0)	1 (0.7)
Foetal disstress	*N* (%)	10 (2.0)	3 (3.8)	7 (2.5)	0 (0.0)	0 (0.0)
Bradycardia	*N* (%)	10 (2.0)	1 (1.1)	6 (2.1)	0 (0.0)	3 (2.1)
Dystocia	*N* (%)	5 (1.0)	3 (3.8)	0 (0.0)	1 (5.9)	1 (0.7)
Further	*N* (%)	80 (15.3)	13 (16.7)	48 (16.9)	2 (11.8)	17 (11.6)
Treatment of birth complications						
Drugs	*N* (%)	78 (30.5)	14 (26.4)	37 (30.3)	3 (37.5)	24 (32.9)
Intubation and ventilation	*N* (%)	75 (29.3)	15 (28.3)	34 (27.9)	2 (25.0)	24 (32.9)
Therapeutic hypothermia treatment	*N* (%)	51 (19.9)	11 (20.8)	27 (22.1)	1 (12.5)	12 (16.4)
Reanimation	*N* (%)	46 (18.0)	11 (20.8)	23 (18.9)	1 (12.5)	11 (15.1)
Coma	*N* (%)	3 (1.2)	0 (0.0)	1 (0.8)	0 (0.0)	2 (2.7)
Phototherapy	*N* (%)	2 (0.8)	2 (3.8)	0 (0.0)	0 (0.0)	0 (0.0)
Reduction of the intracranial pressure	*N* (%)	1 (0.4)	0 (0.0)	0 (0.0)	1 (12.5)	0 (0.0)

#### Birth weight

Evaluation of birth weight without regard to the week of pregnancy the newborns show a trend towards higher birth weights in the group of patients with hypercalcaemia compared with newborns in the group with normocalcaemia (Table 2). The normocalcaemia group was the only one in which birth weights of less than 2000 g were described. In the hypocalcaemia group, birth weights were a median of 1150 g below the total group of newborns (median 3700 g). Overall, *n* = 74 infants had birth weights > 4000 g or were explicitly described as macrosomia. Evaluation of birth weights showed a trend towards higher birth weights in the group of patients with hypercalcaemia (median + 136 g) than in the other two groups of infants (hypo- and normocalcaemia).

#### APGAR score

*N* = 78 (61.4%) of all newborns for whom an APGAR score after the first minute of life was available had a 1-min APGAR of ≤ 3 points, and only *n* = 13 infants (10.2%) had a 1-min APGAR of ≥ 8 points (Table 2). Especially in the group of patients with SCFN and with hypercalcaemia, 68.1% of the newborns (*n* = 47) had APGAR values between 0 and 3 and only 4.3% (*n* = 3) had an APGAR value of ≥ 8. More than 60% of the infants (*n* = 56) showed an APGAR value of ≤ 8 after 10 min.

#### Birth complications

The most frequently described birth complications were asphyxia (*n* = 62, 11.9%), respiratory distress (*n* = 49, 9.4%) and meconium aspiration (*n* = 48, 9.2%). In the group of neonates with SCFN and normocalcaemia, respiratory distress (*n* = 10, 12.8%) dominated, whereas in the group of neonates with SCFN and hypercalcaemia, meconium aspiration (*n* = 35, 12.3%) occurred more frequently. Other complications that has been described were meconium in the amniotic fluid, hypoglycaemia, hypoxic-ischemic encephalopathy and cerebral seizures.

#### Treatment of birth complications

In 30.5% (*n* = 78) of all cases with birth complications and information about the treatment of the birth complications that occurred, drug therapies were initiated. In 29.3% (*n* = 75) of the neonates with birth complication an intubation and ventilation were performed. Therapeutic hypothermia treatment was initiated in 19.9% (*n* = 51) of the neonates. There were no differences in the reported treatments of birth complications between the three groups of calcium levels in infants with SCFN.

#### Amniotic infection/early onset newborn sepsis

In *N* = 22 case reports, early onset sepsis or newborn infection was reported as a complication that occurred during or in the first days after birth in the children. In all cases the infection was present prior to the SCFN.

#### Clinical symptoms

In the group of patients with hypercalcaemia, the typical symptoms of hypercalcaemia such as vomiting (*n* = 25, 12.9%), poor neonatal feeding (*n* = 15, 7.7%), failure to thrive (*n* = 27, 13.9%), lethargy (n=8, 4.1%) and muscular hypotonia (*n* = 8, 4.1%) were reported (Table 1). In contrast, concomitant cardiac disease was most frequently reported in patients with normal calcium levels (*n* = 6, 27.3%). Laboratory chemistry was mainly used to investigate parameters of calcium balance. The detected serum calcium concentrations in the patients with hypercalcaemia (45.6% of all neonates) ranged from 2.59 to 5.34 mmol/l (3.6 ± 0.6 mmol/l) for total calcium and for 1.33–5.60 mmol/l (1.8 ± 1.0 mml/l) for ionised calcium. The diagnosis of concomitant hypercalcaemia was made in a period from 19 days before to 270 days after the first appearance of subcutaneous fat necrosis. The median of the data in terms of the length of time between the appearance of subcutaneous fat necrosis and hypercalcaemia was 10 days. The diagnosis of hypocalcaemia was related to a shorter duration between diagnosis of SCFN and the diagnosis of electrolyte disturbance.

#### Serum vitamin D concentration

Serum vitamin D concentrations were measured exclusively in the group of patients with SCFN and diagnosed hypercalcaemia (with the exception of one patient) (Table 1). 81.8% of the measured 25(OH)D3 concentrations were within the normal range. A 1,25(OH)2D3 deficiency was present in 33.3% of the patients (*n* = 13). An elevation of 1,25(OH)2D3 was described in 46.2% (*n* = 18) of the neonates. For *n* = 28 patients, data on measured serum 1,25(OH)2D3 and parathyroid hormone (PTH) concentrations were reported. Regardless of the level of 1,25(OH)2D3, 89.3% of these patients showed PTH below the reference range. This corresponds to an overall proportion of 18% of all hypercalcaemia patients with a PTH-independent elevation of serum calcium. Only one patient showed an elevation of PTH at several times in the upper normal limit.

#### Serum triglycerides

Serum triglyceride concentrations were reported in *n* = 27 patients (Table 1). 70.4% of them (*n* = 19) showed a hypertriglyceridemia. In 78.9% of the patients with hypertriglyceridemia (*n* = 15), hypercalcaemia occurred simultaneously.

#### Calcifications in different tissues

Calcification in different tissues was described in 21.9% of the neonates (*n* = 70) (Table 1). 47.4% of calcifications were localized renally and 37.1% of the calcifications were localized in the subcutaneous tissues. Patients with calcifications showed higher calcium concentrations than patients without calcifications (3.9 ± 0.6 mmol/l vs. 3.4 ± 0.6 mmol/l) (data not shown). For patients with persistent calcifications serum calcium concentrations of 4.2 ± 0.5 mmol/l were reported. These calcium concentrations were higher than in patients with a temporary calcification. In the group of neonates with normoglycaemia, calcifications were present in affected skin regions, whereas in the group of neonates with hyperglycemia all calcifications occurred outside the necrosis (data not shown).

#### Duration until resolution of symptoms

The median duration of time between appearance and healing of the affected skin lesion showed no differences in infants with SCFN and with diagnosed normo- or hypercalcaemia (Table 2). In the group of neonates with SCFN and with diagnosed hypercalcaemia, the longest disease courses in the amount of 365 days have been described. Complete healing of skin lesions was described for *N* = 136 (42.5%) patients. No information about the course of necrosis were available for *N* = 137 (42.8%) of the neonates with SCFN. 4.1% (*N* = 13) of the neonates with SCFN died.

#### Long-term consequences

In *N* = 137 patients, no information regarding long-term effects were available. Complete healing of skin lesions without other long-term conseqeunces was reported in *N* = 136 case reports. For *n* = 34 patients long-term consequences such as scars on the affected skin sites and calcifications in various tissues have been described. Majority of long-term consequences have been described in the group of infants with concomitant hypercalcaemia. We observed a tendency for greater size of lesions in the group of infants with hypercalcaemia compared to the other groups of infants. The time of the last follow-up in these patients was between 2 months and 10 years of age. Another frequently described long-term consequence was nephrocalcinosis. This episode occurred in *n* = 21 infants with SCFN, and the age of the infants at the last follow-up ranged from 4 months to 15 years. There was no restriction of renal function recorded. Further reported long-term conseqeunces were calcifications of the myocardium (described in *n* = 3 patients) and calcifications at other sites such as the vena cava or in subcutaneous tissue (described in *n* = 2 patients). *N* = 2 patients described persistent movement disorders due to the paresis of the radial nerve and in *n* = 1 patient persistent nephrolithiasis was described.

### Treatment of SCFN-associated complications in neonates

#### Treatment of birth complications

*N* = 78 (30.5%) of cases received medical therapy and *n* = 75 (29.3%) were intubated or ventilated. Therapeutic hypothermia treatment was initiated in *n* = 51 (19.9) neonates, using either a cooling mat, selective head cooling, or ice packs. Furthermore, reanimation had to be initiated in *n* = 46 (18%) of the infants for whom information regarding postnatal therapy was available.

#### Treatment of hypercalcaemia

Intravenous fluid substitution (i.v. hydration) was most frequently reported (in *n* = 73 case reports) to treat the hypercalcaemia in newborns with SCFN. The administration of diuretics has been described for *n* = 72 patients and the administration of glucocorticoids has been described for *n* = 66 patients. Neonatal calcium- and vitamin D-deficient nutrition was started in *n* = 60 neonates with hypercalcaemia. Less frequently, bisphosphonates were administered (*n* = 23). Prophylactic neonatal vitamin D administration was not started or interrupted for the time being (*n* = 14), or various citrates were administered (*n* = 20) (data not shown).

### Maternal risk factors related to SCFN in newborns

#### Maternal and pregnancy parameters

Information about maternal age at time point of delivery or number of pregnancies was available in only *n* = 73 of all case reports (*n* = 320) (Table 3). Mean maternal age at time of delivery of the child was 30.8 ± 6.7 years. On average, it was the 3rd pregnancy (2.9 ± 2.6). In one third of the newborns, diagnosed with SCFN, the presence of gestational diabetes mellitus (GDM) (*n* = 24) or maternal arterial hypertension or preeclampsia (*n* = 19) or diabetes mellitus type 1 (DM1) (*n* = 6) or the intake of various medications or maternal smoking (*n* = 6) were reported as pregnancy characteristics. The presence of GDM or DM1 was higher in the group of neonates with SCFN and with hypercalcaemia than in the group of neonates with normocalcaemia (GDM *n* = 10 vs. (*n* = 3); DM1 *n* = 3 vs. *n* = 1). In *n* = 19 cases, maternal arterial hypertension or preeclampsia was described as pregnancy characteristics.

**Table 3:** Maternal characteristics that are potentially related to the development of SCFN in their newborns

			Subgroups			
		All patients (*n* = 320)	Normocalcaemia (*n* = 52)	Hypercalcaemia (*n* = 146)	Hypocalcaemia (*n* = 9)	No information about calcium level (*n* = 113)
Maternal age at delivery (years)	*N*	73	14	28	1	28
	Mean ± SD	30.8 ± 6.7	31.4 ± 5.8	30.3 ± 5.4	30	30.7 ± 8.05
Gestational diabetes	*N*	24	3	10	1	10
	%	23.3	15.8	33.3	100	32.3
Hypertension/preeclampsia	*N*	19	5	4	0	9
	%	18.4	26.3	13.3	0	29.0
Diabetes mellitus type 1	*N*	6	1	3	0	3
	%	5.8	5.3	10	0	9.7
Maternal smoking	*N*	6	2	2	0	3
	%	5.8	10.5	6.6	0	9.7
Pregnancy duration (weeks)	*N*	117	22	57	2	36
	Mean ± SD	39.3 ± 2.6	38.3 ± 3.7	39.7 ± 2.1	41 ± 0	38.3 ± 3
Mode of delivery	*N*	320	52	146	9	113
Vaginal	*N* (%)	120 (37.5)	24 (46.2)	36 (24.7)	2 (22.2)	58 (51.3)
Sectio	*N* (%)	154 (48.1)	23 (44.2)	83 (56.8)	7 (77.8)	41 (36.3)
No information available	*N* (%)	46 (14.4)	5 (9.6)	27 (18.5)	0 (0.0)	14 (12.4)

#### Duration of pregnancy

Pregnancy duration was reported for *n* = 247 neonates (77%). The average pregnancy duration was 39.3 ± 2.6 weeks (Table 3). 90% of the newborns were born after the 38th week of pregnancy. Ten percent of newborns were born preterm (< 38th week of pregnancy). Newborns without an electrolyte disturbance were born at 38.3 ± 3.7 weeks of pregnancy, whereas newborns who developed hypercalcaemia during the disease course were born at 39.7 ± 2.1 weeks of pregnancy.

#### Type of delivery

While in the group of newborns with SCFN and normocalcaemia, vaginal delivery and section were performed with equal frequencies (*n* = 24 vs. *n* = 23), in the group of newborns with SCFN and with hypercalcaemia there was a significantly higher proportion of section (*n* = 83, 56.8%) compared to vaginal delivery (*n* = 36, 24.7%) (Table 3). The main reasons for a cesarean section were foetal distress (58.3%, *n* = 60) and failure to progress in the birth process (14.6%, *n* = 15).

## Discussion

The aim of this systematic review was to identify published cases of diagnosed SCFN in newborns in the literature, to summarise the described clinical feature, to identify risk factors for the development of SCFN and to summarise hypotheses of pathophysiology. We screened *n* = 2.255 publications. *N* = 206 publications were finally included in our analysis. In these, *n* = 320 cases of newborns with SCFN were described. We have observed that (1) there were no gender differences in diagnosis of SCFN in newborns, (2) the typical skin lesions of SCFN appear usually 1 week after birth and were localized especially on the back, shoulders, cheeks and proximal limbs, (3) in most of the cases of newborns with SCFN, mothers reported GDM, hypertension or preeclampsia during pregnancy, (4) approximately 10 days after the presence of typical skin lesion of SCFN, altered serum calcium concentrations were described and 45.6% of the newborns with SFCN developed a hypercalcaemia (independent from PTH in 18% of the cases), (5) most of the skin lesions healed without complications within 3 months, (6) in those newborns with SCFN and with hypercalcaemia it has been observed that there was a longer duration of pregnancy, a higher birthweight, bigger skin lesions, more concomitant symptoms and a longer course of disease compared to newborns with SCFN and with normal serum calcium levels, (8) in 14.7% of the newborns with SCFN long-term consequences have been described.

### Gender differences in diagnosis of SCFN

Our observation of equal distribution of sex in newborns diagnosed with SCFN is consistent with previous findings from literature [18, 19]. This observation indicated a sex-independent pathophysiological mechanism for the development of SCFN.

### Relationship between maternal metabolic factors and SCFN in newborns

In the majority of the published cases of newborns with SCFN, the mother reported GDM, hypertension or preeclampsia during pregnancy. This is in line with literature, where it has been shown that the pregnancy metabolic complications such as GDM and hypertension or preeclampsia were significantly more frequent in mothers of children with SCFN compared to the total complications of pregnancy in the normal population (GDM 14.4 vs. 5.4%, hypertensive pregnancy diseases: 18.2 vs. ≤ 8%) [20, 21].

As a consequence of GDM, the fetus experiences an increased insulin secretion. This can lead to an increase in the amount of foetal adipose tissue and thus in an increase in the birth weight [22]. In the presented systematic review, *n* = 74 newborns (33.9%) with later diagnosed SCFN had birth weights > 4000 g or were explicitly described as macrosomia. This is in line with literature, where it has been described that the incidence of macrosomia in neonates with SCFN was higher than in the general population (33.9 vs. < 10%) [23, 24]. It is assumed that a birth weight > 4000 g is related to an increased mechanical pressure at birth and an associated hypoperfusion of the tissue [25].

It is supposed that a placental insufficiency, caused by hypertensive pregnancy complications, is the link between hypertension or preeclampsia during pregnancy and the development of SCFN in newborns. Placental insufficiency is associated with an increased risk of poorer foetal outcome, which includes a higher risk for the occurrence of birth complications [8, 26].

### Relationship between birth complications and SCFN

**Fig. 3:**
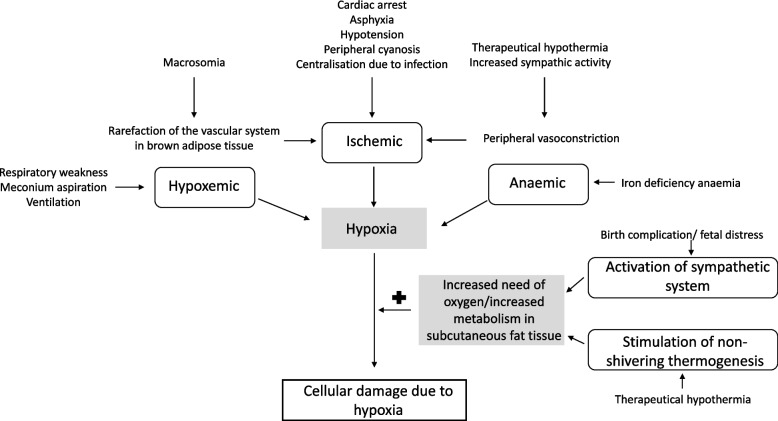
Overview of possible reasons of hypoxia and an increased need of oxygen or an increased metabolism of subcutaneous fat necrosis leading to a cellular damage in subcutaneous fat tissue

42.2% of the described birth complications in newborns with SCFN (asphyxia, respiratory weakness, HIE, hypoxaemia, infection or sepsis, peripheral cyanosis, hypotension, anaemia, foetal distress, bradycardia) were related to peripheral tissue hypoxia. For 37.9% of the newborns with SCFN it has been described that a therapeutic intervention in the form of resuscitation and therapeutic hypothermia after birth was conducted. It is assumed that a peripheral oxygen depletion occurred in these infants because of the indication for the intervention or because of the intervention itself. It is hypothesised that an oxygen depletion leads to hypoxia and that a hypoxic cell damage promotes the necrosis in skin lesions [2]. Figure 3 summarizes the possible causes and influences leading to the hypoxic cell damage in the context of SCFN in the newborn.

### Localisation of SCFN in newborns

In the case reports evaluated in the present review, the characteristic skin lesions of subcutaneous adipose tissue necrosis were most frequently on the back, buttocks, cheeks and proximal extremities. It is hypothesised that there is a relationship between the location of SCFN and the areas in which primary brown adipose tissue is localized in neonates. In the newborn, brown adipose tissue is localized interscapular (diamond-shaped), in the neck, infraclavicular and axillary, along the intercostal arteries, esophageal and tracheal, around large vessels, surrounding the kidneys and adrenal glands, and on the posterior abdominal wall [27–29]. Ichimiya et al. examined biopsy material from two children with SCFN from the affected skin sites. Immunohistochemical staining with antibodies against UCP-1, a protein specific for brown adipose tissue, demonstrated the presence of brown adipose tissue within the necrotic tissue [5]. But within the here presented systematic review, the affected skin areas of SCFN were only in 34.5% of all cases in areas for which the presence of brown adipose tissue has been described.

### Age of onset and size of skin lesion typical for SCFN in newborns and duration until electrolyte disturbances were detected

For the majority of newborns with SCFN the presence of typical skin lesions has been described within the first week of birth. The presence of typical skin lesions for SCFN on a later time point has only been reported in newborns with SCFN and with diagnosed hypercalcaemia. The latest time point of presence of typical skin lesions for SCF has been reported at day 70 after birth. In 98% of the newborns with SCFN the associated electrolyte disturbance occurred within a period of 5 months. This observation is in line with a previous work, which described that electrolyte alterations in newborns with SCFN were obtained within the first 6 months [30]. In 70.5% of the newborns with SCFN and for which serum calcium levels have been reported, a hypercalcaemia has been described. In previous case reports and reviews of cases of newborns with SCFN, the frequency of hypercalcaemia has been reported in the range between 25 and 69% [18, 19]. The diagnosis of hypercalcaemia was described at a median of 10 days after the presence of typical skin lesion of SCFN. Since the presence of a hypercalcaemia is rare in neonates [31], this electrolyte disturbance seems to be related to SCFN in newborns. Interestingly, the time between the presence of typical skin lesions of SCFN and the diagnosis of an altered serum calcium level was shorter in the group of patients with SCFN and hypocalcaemia than in the group of patients with SCFN and hypercalcaemia. The evaluation of the size of the skin lesions showed no correlation with serum calcium levels newborns with SCFN. However, there is a tendency for larger lesions in the group of patients with hypercalcaemia compared to all other patients. Furthermore, we observed that the presence of a hypercalcaemia in newborns with SCFN is related to prolonged course of the disease, compared to newborns with SCFN and normal serum calcium levels. One hypothesis is, that this prolonged disease course is related to the tendency of larger skin lesions in newborns with SCFN and hypercalcaemia. It is assumed, that the size of skin lesion correlates with the extent of inflammation and the resulting immune response [10, 32, 33]. Since we observed in a subgroup of patients with reported levels of serum 1,25(OH)2D3 and parathyroid hormone (PTH) that the majority of these patients showed a suppressed PTH level, independent of serum 1,25(OH)2D3 levels, a PTH-independent but immune-stimulated hypercalcaemia is assumed.

### Relationship between serum calcium level and calcifications in newborns with SCFN

In the presented systematic review, *n* = 70 newborns with SCFN showed calcifications, of which 84.3% were in the subgroup of newborns with SCFN and hypercalcaemia. 37.1% of the described calcifications were directly in the necrotic skin area and 47.4% were present in form of nephrocalcinosis/nephrolithiasis. Calcifications that occurred outside subcutaneous tissue were found exclusively in the group of newborns with SCFN and diagnosed hypercalcaemia. 58.7% of renal calcifications were still persistent at the last follow-up visits, without concomitant impairment of renal function. Retrospectively, patients with persistent nephrocalcinosis had the highest serum calcium levels. Calcifications in various tissues are a frequent complication of hypercalcaemia [34]. We assume that calcifications in subcutaneous tissues arise primarily from a local process that is probably triggered by the necrosis itself.

### Relationship between the duration of the symptoms of SCFN and serum calcium levels

Within the presented systematic review, we observed that the duration of the symptoms of SCFN ranged between 14 and 365 days. We identified a dependency of duration of symptoms from measured serum calcium levels in newborns with SCFN. The duration of symptoms of SCFN was similarly in the group of newborns with SCFN with normal serum calcium levels and those with a hypocalcaemia, but was longer in newborns with SCFN and diagnosed hypercalcaemia. The longest described duration of symptoms in the amount of 365 days has been described in a patient with SCFN associated hypercalcaemia [35]. It is assumed that the presence of a hypercalcaemia in newborns with SCFN is a risk factor for a longer course of the disease. As we described before, newborns with SCFN and diagnoses hypercalcaemia tend to larger skin lesions. We hypothesise that a longer duration of symptoms in these patients could related to the larger size of skin lesion.

### Therapy of SCFN-associated hypercalcaemia

Sixty percent of the newborns with SCFN with diagnosed hypercalcaemia were treated by i.v. fluid substitution, administration of loop diuretics and glucocorticoids. Other patients received a low-calcium/low-vitamin D diet. In 20% of the newborns with SCFN with diagnosed hypercalcaemia a bisphosphonate therapy was introduced. This form of therapy has primarily described for newborns with a severe form of hypercalcaemia [36–38]. To sum up, the treatment of a hypercalcaemia in neonates includes a low calcium/vitamin D diet, discontinuation of calcium/vitamin D, i.v. hydration and the use of loop diuretics (e.g. furosemide). In addition, glucocorticoids or bisphosphonates can be used [31].

### Long-term consequences of SCFN

Long-term consequences have been identified in this systematic review in one of four newborns with SCFN. These long-term consequences include scars at the affected skin sites and persistent nephrocalcinosis. *N* = 13 infants died during the course of the disease, but in 60% of these the cause of death was not related to SCFN and in the remaining *n* = 5 infants, the cause of death remained unclear. However, it must always be taken into account when interpreting these observations that persistent calcifications are associated with the higher serum calcium concentrations in newborns within the course of the disease. As we have discussed in the section before, an early identification of altered serum calcium levels and the initiation of the treatment of increased serum calcium level may reduce the risk of persistence calcifications. The results of this work suggest that calcifications in subcutaneous tissues are mainly due to a local process that is probably triggered by necrosis itself. However, calcifications outside the subcutaneous tissue occurred only when there was a systemically effective elevation of serum calcium levels.

### Discussion of possible pathomechanisms for the development of the SCFN in newborns

#### Mechanical compression as a cause of SCFN

In individual cases, skin lesions at sites that could be associated with a local traumatic event have been described in the literature [4]. In addition, shoulder dystocia during delivery was reported in several infants reported and their skin lesions subsequently appeared in the neck, shoulders, axilla, sites that may be consistent with the presence of shoulder dystocia [39–41]. However, because subcutaneous adipose tissue necrosis also occurs in infants without shoulder dystocia at the same sites, no causal relationship between a possible mechanical compression and the occurrence of the disease.

#### Therapeutic hypothermia as a cause of SCFN in newborns

In the literature, the implementation of therapeutic hypothermia in newborns is discussed as an important risk factor for the development of SCFN [14, 42]. During therapeutic hypothermia, the body temperature is lowered to a target temperature of 33.5 ± 0.5 °C (rectal) for a period of 72 h [33]. As a result of the physiological response to a cold stimulus, blood flow is redirected from the periphery to the vital organs [16, 17, 43]. It is assumed that the associated hypoxia in the skin leads to necrosis of adipocytes [14, 42]. The maintenance of this metabolism can only be ensured by an increase in perfusion. But it remains questionable whether the fall in temperature alone is sufficient to induce a great difference in oxygen demand and perfusion that is related to hypoxic cell damage in the adipocytes. We assumed rather that there is an interplay with other risk factors for the development of SCFN, such as the birth complications or macrosomia at birth.

Furthermore, an important role of the brown adipose tissue is discussed. Both, in animal models and in humans, it has been shown, that an exposure to a cold environment is related to an activation of thermogenesis in brown adipose tissue [27, 44, 45], to an increased glucose uptake into the brown adipocytes [46, 47], to an increased oxygen demand [12], and to an increase in perfusion [46, 48]. These findings suggest that the metabolism within the brown adipose tissue increases with a decrease in external temperature. In contrast, only in 34.5% of all patients SCFN appeared in locations, where brown adipose tissue has been detected before.

Furthermore, it is assumed that the use of therapeutic hypothermia is associated with a resolution of crystallisation of fat within adipocytes. A higher melting point of the subcutaneous adipose tissue of the neonate compared to adults due to the altered fatty acid composition is assumed to increase the risk of crystallisation. The adipose tissue of the newborn has a higher proportion of saturated fatty acids compared with the adipose tissue of adults [49, 50]. It is assumed that a lowering of the temperature, for example by therapeutic hypothermia in the subcutaneous adipose tissue is related to an easier crystallisation of the neonatal adipose tissue due to the higher melting point. The development of the melting temperature of fat was also studied by Channon and Harrison [3]. They showed an decrease of the melting temperature of fat with increasing age [3]. They also compared the adipose tissue of children with sclerema neonatorum, a disease which also belongs to the group of panniculitides, with adipose tissue of healthy children of the same age. Thereby, the children with sclerema neonatorum showed a higher melting temperature and a higher content of saturated fatty acids in the adipose tissue than healthy children of the same age [3]. Comparing the data of a neonate with SCFN with the data on fatty acid proportions in subcutaneous adipose tissue in adults [51], the diseased neonate shows a significantly higher proportion of saturated palmitic acid (48.4% versus 21.4% in adults) and a significantly lower proportion of unsaturated oleic acid (29.1% versus 38.2% in adults). The observations from this case report support the notion that there may be a relationship between the composition of neonatal subcutaneous adipose tissue and the development of the disease.

### Discussion of pathomechanisms for the development of a hypercalcaemia in the presence of SCFN in newborns

#### Extrarenal vitamin D formation as a cause of hypercalcaemia

Within the presented systematic literature review, we observed that 89.3% of newborns with SCFN and with reported data on serum 1,25(OH)2D3 and on parathyroid hormone (PTH) had a suppressed PTH level, independent of the serum 1,25(OH)2D3 level. A PTH-independent hypercalcaemia is assumed. Within literature an extrarenal 1,25(OH)2D3 formation as a trigger for the elevation of serum calcium levels in newborns with SCFN is discussed [10, 32, 52]. The formation of the active 1,25(OH)2D3 (calcitriol) from its biologically inactive form 25OHD3 (=calcidiol) occurs by hydroxylation by the enzyme 25-hydroxyvitamin D3-1α-hydroxylase (1α-hydroxylase). The enzyme 1α-hydroxylase belongs to the group of CYP-450 enzymes [37] and is primarily expressed in the epithelial cells of the proximal renal tubule [6, 53, 54]. The physiological function and regulation of renal 1α-hydroxylase is characterised by the PTH-dependent stimulation and by a negative feedback mechanism through its own reaction product, 1,25(OH)2D3. This negative feedback mechanism is thereby mediated via nuclear vitamin D receptors (VDR) [55]. Since subcutaneous adipose tissue necrosis presents both, the histological picture of a granulomatous disease and a high risk of hypercalcaemia, a similar pathomechanism as in other granulomatous diseases (e.g. sarcoidosis or tuberculosis) is assumed [53]. Extrarenal expression of 1α-hydroxylase has been demonstrated in various patients with granulomatous diseases [7, 56, 57]. Farooque et al. [52] investigated the expression of 1α-hydroxylase in skin samples from two patients with histologically confirmed diagnosis of SCFN. He was able to demonstrate very strong expression of 1α-hydroxylase in the inflammatory infiltrate of subcutaneous adipose tissue necrosis in both cases. A crucial role seems to be played by the lack of feedback mechanisms in peripheral macrophages, triggered by the antagonistic effect of γ-interferon on 1,25(OH)2D3 effects at the level of mRNA (messenger RNA) synthesis in cells [26, 58]. The peripheral form of 1α-hydroxylase is mainly subject to immunoregulatory processes through IFN- γ and IL-2. Both IFN- γ and IL-2 contribute as cytokines to the development of granulomatous reactions by stimulating cell differentiation from macrophages to epithelioid and foreign body giant cells [59]. For many years, the literature has considered an exuberant reaction with an accumulation of T helper cells as a possible cause of sarcoidosis [60]. Ramstein et al. [61] recently described an increased transformation of Th17 cells to Th17.1 cells. They also demonstrated that Th17.1 cells are a major source of IFN-γ produced in sarcoidosis patients. It is assumed that also in patients with SCFN, the specific differentiation of certain T helper cells serves as a source of IFN-γ, leading to stimulation of peripheral 1α-hydroxylase.

The degradation of 1,25(OH)2D3 occurs by the enzyme 24α-hydroxylase to its inactive metabolite 24,25(OH)2D. This reaction step is stimulated by the increase in 1,25(OH)2D3, ensuring a constant 1,25(OH)2D3 level in plasma [56]. Bahadur et al. [32] observed elevated 1,25(OH)2D3 levels in their patient with SCFN concomitant with low levels of the inactive degradation product 24,25(OH)2D. They concluded an impaired degradation by the enzyme 24α-hydroxylase, which is encoded by the gene CYP2A41 [62]. The observations made in this patient support the findings of Vidal, Ramana and Dusso [63] who described the mechanism of decreased transcription of 24α-hydroxylase. It seems that there is a direct protein-protein interaction between the transcription factor STAT1 and the vitamin D receptor, preventing binding to the associated vitamin D response element (VDRE) and, as a consequence, no transcription of 24α-hydroxylase [63]. Assuming that the enzymatic regulatory mechanisms within the vitamin D balance is the same in SCFN as in other granulomatous diseases, it can be assumed that the increase in serum 1,25(OH)2D3 levels is the result of increased expression of the peripheral form of 1α-hydroxylase with concomitant decreased expression of peripheral 24α-hydroxylase.

#### Direct calcium release from the necrotic skin areas

Several authors suggest that in newborns with SCFN and associated hypercalcaemia, there is a direct release of calcium from the necrotic skin areas [9, 43, 63]. Due to the results of this review that there could not be found a correlation between the size of skin lesion and serum calcium level, this hypothesis does not seem to be the main cause of hypercalcaemia.

### Methodological strengths of the work

A strength of this work lies in the systematic execution of the literature search following the guidelines of the PRISMA statement [64]. In the selection of literature databases, emphasis was placed on a close reference and the largest possible collection of publications on medical topics in order to obtain the largest possible number of hits. Thus, the search could be reproduced in *n* = 5 of the *n* = 6 databases searched. However, the use of the online search engine Google-Scholar cannot be compared to the other databases in terms of reproducibility, as search algorithms may change here over time, leading to different results. Because of the size of this database and the goal of identifying all case reports published to date, the online search engine was included despite its weakness. Regarding the search terms used, it should be noted that their selection before the actual search procedure may have resulted in reduced sensitivity. Thus, it cannot be ruled out that a limitation of the number of hits resulted from the search terms used and the application of a Boolean operator. Another strength of this study is the comparison of a large patient collective. In previous literature, only case series of up to *n* = 17 patients have been published. Thus, the amount of data generated here is clearly superior to previous publications in this regard and may thus provide new insights. Furthermore, no comparison of data depending on concomitant electrolyte disturbance has been published in the literature so far. The division into subgroups depending on the reported serum calcium level performed here aimed to identify possible risk factors favouring the occurrence of such an electrolyte disturbance. The systematic literature search was followed by the selection of studies to be included in this work. Two consecutive screening steps were performed. This was to ensure the most specificity of the included studies was achieved.

## Limitations

We assumed that not all available clinical data of newborns with SCFN were published by the authors. As a result, for some parameters there was only a small data set available for analysis. Furthermore, the comparability of published laboratory data in newborns with SCFN is a critical issue, since there are differences in the measurement method.

## Conclusion

Within this systematic literature review, we identified maternal risk factors that are related to the development of SCFN in newborns. These risk factors include a maternal GDM, hypertension or preeclampsia during pregnancy as well as therapeutic hypothermia treatment. Furthermore, we summed up the hypotheses to suggest a pathophysiological model for the development of SCFN in newborns. It is assumed that a hypoxic cell damage could be related to the development of SCFN in newborns. It is hypothesised, that there is an imbalance of cellular oxygen demand and the amount of available oxygen in the subcutaneous adipose tissue. This imbalance is associated with prenatal risk factors, birth complications and therapeutic interventions. Some newborns with SCFN were diagnosed with hypercalcaemia. Within this systematic literature review we identified risk factors that may serve as hints in the clinical course of the SCFN and are helpful to rapidly identify hypercalcaemia and thus treat it before the onset of concomitant symptoms. An extrarenal synthesis of Vitamin D, as it is known from granulomatous diseases, seems to be the reason of hypercalcaemia in SCFN. Although SCFN in newborns is considered a benign, self-limited condition, it can have dangerous extracutaneous side effects. Clinicians should know risk factors related to SCFN in newborns and should monitor newborns with SCFN to avoid the risk of serious complications, with particular reference to hypercalcaemia.

## Supplementary Information

**Additional file 1: Table S1.** Included publication in analysis of the research question. **Table S2.** Excluded references and reason for exclusion.

## Data Availability

The datasets generated and analysed during the current study are available from the corresponding author on reasonable request.

## Abbreviations

APGAR: Appearance, Pulse, Grimace, Activity und Respiration
SCFN: Subcutaneous fat necrosis in newborns
GDM: Gestational diabetes mellitus
DM1: Diabetes mellitus type 1
HIE: Hypoxic-ischemic encephalopathy
PTH: Parathyroid hormone
25OHD3: Calcidiol
1,25(OH)2D3: Calcitriol
VDR: Nuclear vitamin D receptor

## References

[1] Requena L, Yus S, E: Panniculitis. Part II. (2001). Mostly lobular panniculitis. J Am Acad Dermatol.

[2] Holzel A (1951). Subcutaneous fat necrosis of the newborn. Arch Dis Child.

[3] Channon HJ, Harrison GA (1926). The chemical nature of the subcutaneous fat in the normal and sclerematous infant. Biochem J.

[4] Hicks MJ, Levy ML, Alexander J, Flaitz CM (1993). Subcutaneous fat necrosis of the newborn and hypercalcemia: case report and review of the literature. Pediatr Dermatol.

[5] Ichimiya H, Arakawa S, Sato T, Shimada T, Chiba M, Soma Y, Mizoguchi M, Tomonari K, Iwasaka H, Hatano Y (2011). Involvement of brown adipose tissue in subcutaneous fat necrosis of the newborn. Dermatology.

[6] Adams JS, Gacad MA (1985). Characterization of 1 alpha-hydroxylation of vitamin D3 sterols by cultured alveolar macrophages from patients with sarcoidosis. J Exp Med.

[7] Cadranel J, Hance AJ, Milleron B, Paillard F, Akoun GM, Garabedian M (1988). Vitamin D metabolism in tuberculosis. Production of 1,25(OH)2D3 by cells recovered by bronchoalveolar lavage and the role of this metabolite in calcium homeostasis. Am Rev Respir Dis.

[8] Glover MT, Catterall MD, Atherton DJ (1991). Subcutaneous fat necrosis in two infants after hypothermic cardiac surgery. Pediatr Dermatol.

[9] Sharlin DN, Koblenzer P (1970). Necrosis of subcutaneous fat with hypercalcemia. A puzzling and multifaceted disease. Clin Pediatr (Phila).

[10] Tran JT, Sheth AP (2003). Complications of subcutaneous fat necrosis of the newborn: a case report and review of the literature. Pediatr Dermatol.

[11] Flemmer AW, Maier RF, Hummler H (2013). S2k-Leitlinie: Behandlung der neonatalen Asphyxie unter besonderer Berücksichtigung der therapeutischen Hypothermie.

[12] Dawes GS, Lewis BV, Milligan JE, Roach MR, Talner NS (1968). Vasomotor responses in the hind limbs of foetal and new-born lambs to asphyxia and aortic chemoreceptor stimulation. J Physiol.

[13] Irving L, Scholander PF, Grinnell SW (1942). The regulation of arterial blood pressure in the seal during diving. Am J Physiol.

[14] Cheung PY, Gill RS (2011) Bigam DL: A swine model of neonatal asphyxia. J Vis Exp 11(56):3166 10.3791/3166 PMC3227176 22006174

[15] Gekle M, Wischmeyer E, Gründer S, Petersen M, Schwab A, Thieme (2010). Taschenlehrbuch Physiologie.

[16] Lopez M, Sessler DI, Walter K, Emerick T, Ozaki M (1994). Rate and gender dependence of the sweating, vasoconstriction, and shivering thresholds in humans. Anesthesiology.

[17] Yanagisawa O, Homma T, Okuwaki T, Shimao D, Takahashi H (2007). Effects of cooling on human skin and skeletal muscle. Eur J Appl Physiol.

[18] Burden AD, Krafchik BR (1999). Subcutaneous fat necrosis of the newborn: a review of 11 cases. Pediatr Dermatol.

[19] Mahé E, Girszyn N, Hadj-Rabia S, Bodemer C, Hamel-Teillac D, De Prost Y (2007). Subcutaneous fat necrosis of the newborn: a systematic evaluation of risk factors, clinical manifestations, complications and outcome of 16 children. Br J Dermatol.

[20] De la Torre-Gutiérrez M, Padilla-Muñoz H, Pérez Rulfo-Ibarra D, Castillo-Villarruel F, Angulo-Castellanos E, Campos-Sierra A, Barrera-Sánchez F, Stanley-Lucero MA, Alfaro-Castellanos DE, García-Magdaleno PE, et al (2014) Fat necrosis in a newborn. Case Report. Rev Med MD 14(1):248–250

[21] Zhu Y, Zhang C (2016). Prevalence of gestational diabetes and risk of progression to type 2 diabetes: a global perspective. Curr Diab Rep.

[22] Kc K, Shakya S, Zhang H (2015). Gestational diabetes mellitus and macrosomia: a literature review. Ann Nutr Metab.

[23] Akin Y, Cömert S, Turan C, Piçak A, Ağzikuru T, Telatar B (2010). Macrosomic newborns: a 3-year review. Turk J Pediatr.

[24] Foster DO, Frydman ML (1979). Tissue distribution of cold-induced thermogenesis in conscious warm- or cold-acclimated rats reevaluated from changes in tissue blood flow: the dominant role of brown adipose tissue in the replacement of shivering by nonshivering thermogenesis. Can J Physiol Pharmacol.

[25] Collins KA, Popek E (2018). Birth injury: birth asphyxia and birth trauma. Acad Forensic Pathol.

[26] Mustafa R, Ahmed S, Gupta A, Venuto RC (2012). A comprehensive review of hypertension in pregnancy. J Pregnancy.

[27] Aherne W, Hull D (1966). Brown adipose tissue and heat production in the newborn infant. J Pathol Bacteriol.

[28] Haider S (2012) Images in paediatrics: subcutaneous fat necrosis causing radial nerve palsy. BMJ Case Rep 10:bcr1020114904 10.1136/bcr.10.2011.4904 PMC4543001 22665708

[29] Hu HH, Tovar JP, Pavlova Z, Smith ML, Gilsanz V (2012). Unequivocal identification of brown adipose tissue in a human infant. J Magn Reson Imaging.

[30] Onyiriuka AN, Utomi TE (2017). Hypocalcemia associated with subcutaneous fat necrosis of the newborn: case report and literature review. Oman Med J.

[31] Rodd C, Goodyer P (1999). Hypercalcemia of the newborn: etiology, evaluation, and management. Pediatr Nephrol.

[32] Bahadur KA, Johnson S, Lentzner B, Gangat M, Carlson J, Balachandar S (2018). Hypercalcemia, hyperkalemia and supraventricular tachycardia in a patient with subcutaneous fat necrosis. J Pediatr Endocrinol Metab.

[33] Finne PH, Sanderud J, Aksnes L, Bratlid D, Aarskog D (1988). Hypercalcemia with increased and unregulated 1,25-dihydroxyvitamin D production in a neonate with subcutaneous fat necrosis. J Pediatr.

[34] Kumar V, Abbas AK, Aster JC (2014) Necrosis, mechansims of cell injurys & granulomatous inflammation. In: Kumar V, Abbas AK, Aster JC(H) (eds) Robbins & Cotran Pathologic Basis of Disease E-Book 9 Aufl Elsevier Health Sciences, Philadelphia, pp 31–68 98-99

[35] Beuzeboc Gérard M, Aillet S, Bertheuil N, Delliere V, Thienot S, Watier E (2014). Surgical management of subcutaneous fat necrosis of the newborn required due to a lack of improvement: a very rare case. Br J Dermatol.

[36] Alos N, Chabot G (2008). Importance of the renal calcium load as putative predictor for nephrocalcinosis in subcutaneous fat necrosis associated with severe hypercalcemia. Horm Res.

[37] Hewison M, Zehnder D, Bland R, Stewart PM (2000). 1alpha-Hydroxylase and the action of vitamin D. J Mol Endocrinol.

[38] Kellar A, Tangtatco JA, Weinstein M, Saunders N (2018). Subcutaneous fat necrosis of the newborn with initial hypocalcemia and familial recurrence: a case report. J Cutan Med Surg.

[39] Kwon HS, Lee JH, Kim GM, Bae JM (2017). Image gallery: subcutaneous fat necrosis of the newborn. Br J Dermatol.

[40] Martins J, Maxaud A, Bah AG, Prophette B, Maillard H, Bénéton N (2015). Subcutaneous fat necrosis after moderate therapeutic hypothermia in two newborns of African origin. Arch Pediatr.

[41] Rubin G, Spagnut G, Morandi F, Valerio E, Cutrone M (2015). Subcutaneous fat necrosis of the newborn. Clin Case Rep.

[42] Landau Y, Berger I, Marom R, Mandel D, Ben Sira L, Fattal-Valevski A, Peylan T, Levi L, Dolberg S, Bassan H (2011). Therapeutic hypothermia for asphyxiated newborns: experience of an Israeli tertiary center. Isr Med Assoc J.

[43] Fuchs F, Bouyer J, Rozenberg P, Senat MV (2013). Adverse maternal outcomes associated with fetal macrosomia: what are the risk factors beyond birthweight?. BMC Pregnancy Childbirth.

[44] Hiroshima Y, Yamamoto T, Watanabe M, Baba Y, Shinohara Y (2018). Effects of cold exposure on metabolites in brown adipose tissue of rats. Mol Genet Metab Rep.

[45] Smith DL, Yang Y, Hu HH, Zhai G, Nagy TR (2013). Measurement of interscapular brown adipose tissue of mice in differentially housed temperatures by chemical-shift-encoded water-fat MRI. J Magn Reson Imaging.

[46] Orava J, Nuutila P, Lidell ME, Oikonen V, Noponen T, Viljanen T, Scheinin M, Taittonen M, Niemi T, Enerbäck S (2011). Different metabolic responses of human brown adipose tissue to activation by cold and insulin. Cell Metab.

[47] Virtanen KA, Lidell ME, Orava J, Heglind M, Westergren R, Niemi T, Taittonen M, Laine J, Savisto NJ, Enerbäck S (2009). Functional brown adipose tissue in healthy adults. N Engl J Med.

[48] Forsum E, Löf M, Olausson H, Olhager E (2006). Maternal body composition in relation to infant birth weight and subcutaneous adipose tissue. Br J Nutr.

[49] Sanjurjo P, Aldámiz-Echevarría L, Prado C, Azcona I, Elorz J, Prieto JA, Ruiz JI, Rodríguez-Soriano J (2006). Fatty acid composition of skeletal muscle and adipose tissue in Spanish infants and children. Br J Nutr.

[50] Cevik G, Beken S, Aydin B, Dilli D, Zenciroglu A, Okumus N (2013). Subcutaneous fat necrosis during hypothermia treatment in an asphyxiated infant. Gazi Med J.

[51] Pezeshkian M, Noori M, Najjarpour-Jabbari H, Abolfathi A, Darabi M, Darabi M, Shaaker M, Shahmohammadi G (2009). Fatty acid composition of epicardial and subcutaneous human adipose tissue. Metab Syndr Relat Disord.

[52] Farooque A, Moss C, Zehnder D, Hewison M, Shaw NJ (2009). Expression of 25-hydroxyvitamin D3-1alpha-hydroxylase in subcutaneous fat necrosis. Br J Dermatol.

[53] Fais S, Burgio VL, Silvestri M, Capobianchi MR, Pacchiarotti A, Pallone F (1994). Multinucleated giant cells generation induced by interferon-gamma. Changes in the expression and distribution of the intercellular adhesion molecule-1 during macrophages fusion and multinucleated giant cell formation. Lab Invest.

[54] Liu PT, Stenger S, Li H, Wenzel L, Tan BH, Krutzik SR, Ochoa MT, Schauber J, Wu K, Meinken C (2006). Toll-like receptor triggering of a vitamin D-mediated human antimicrobial response. Science.

[55] Murayama A, Takeyama K, Kitanaka S, Kodera Y, Kawaguchi Y, Hosoya T, Kato S (1999). Positive and negative regulations of the renal 25-hydroxyvitamin D3 1alpha-hydroxylase gene by parathyroid hormone, calcitonin, and 1alpha,25(OH)2D3 in intact animals. Endocrinology.

[56] Karakelides H, Geller JL, Schroeter AL, Chen H, Behn PS, Adams JS, Hewison M, Wermers RA (2006). Vitamin D-mediated hypercalcemia in slack skin disease: evidence for involvement of extrarenal 25-hydroxyvitamin D 1alpha-hydroxylase. J Bone Miner Res.

[57] Zehnder D, Bland R, Williams MC, McNinch RW, Howie AJ, Stewart PM, Hewison M (2001). Extrarenal expression of 25-hydroxyvitamin d(3)-1 alpha-hydroxylase. J Clin Endocrinol Metab.

[58] Dusso AS, Kamimura S, Gallieni M, Zhong M, Negrea L, Shapiro S, Slatopolsky E (1997). gamma-Interferon-induced resistance to 1,25-(OH)2 D3 in human monocytes and macrophages: a mechanism for the hypercalcemia of various granulomatoses. J Clin Endocrinol Metab.

[59] Stoffels K, Overbergh L, Giulietti A, Verlinden L, Bouillon R, Mathieu C (2006). Immune regulation of 25-hydroxyvitamin-D3-1alpha-hydroxylase in human monocytes. J Bone Miner Res.

[60] Wahlström J, Katchar K, Wigzell H, Olerup O, Eklund A, Grunewald J (2001). Analysis of intracellular cytokines in CD4+ and CD8+ lung and blood T cells in sarcoidosis. Am J Respir Crit Care Med.

[61] Ramstein J, Broos CE, Simpson LJ, Ansel KM, Sun SA, Ho ME, Woodruff PG, Bhakta NR, Christian L, Nguyen CP (2016). IFN-γ-producing T-helper 17.1 cells are increased in sarcoidosis and are more prevalent than T-helper type 1 cells. Am J Respir Crit Care Med.

[62] Simonds WF (2012). A new look at vitamin D metabolism and “idiopathic” hypercalcemia. J Clin Endocrinol Metab.

[63] Vidal M, Ramana CV, Dusso AS (2002). Stat1-vitamin D receptor interactions antagonize 1,25-dihydroxyvitamin D transcriptional activity and enhance stat1-mediated transcription. Mol Cell Biol.

[64] Moher D, Liberati A, Tetzlaff J, Altman DG (2009). Preferred reporting items for systematic reviews and meta-analyses: the PRISMA statement. PLoS Med.

