# Supplementary material for: Subcutaneous fat necrosis in newborns: a systematic literature review of case reports and model of pathophysiology
Source: Mol Cell Pediatr. 2022 Nov 24;9:18. doi: 10.1186/s40348-022-00151-1 (PMC9700527; doi:10.1186/s40348-022-00151-1)
Supplement: Supplementary file 1 — Additional file 1: Table S1. Included publication in analysis of the research question. Table S2. Excluded references and reason for exclusion. [file 40348_2022_151_MOESM1_ESM.docx]

**Supplement**

**Tabele S1:** Included publication in analysis of the research question

|  | **ID per case** | **Reference** |
| --- | --- | --- |
| 1 | 2 | Bahadur KA, Johnson S, Lentzner B, Gangat M, Carlson J, Balachandar S: Hypercalcemia, hyperkalemia and supraventricular tachycardia in a patient with subcutaneous fat necrosis. Journal of pediatric endocrinology & metabolism 31: 469–472 (2018) |
| 2 | 3 | Bharani V, Kakkar N, Mahajan V, Azad C: Subcutaneous Fat Necrosis of Newborn: Unfamiliar Histomorphology of a Familiar Lesion. International journal of surgical pathology 26: 621-622 (2018) |
| 3 | 4 | Heskin L, Khan MAA, Ho PH, Burge T: Subcutaneous fat necrosis requiring plastic surgical intervention in an infant treated with whole-body cooling. JPRAS Open 15: 61–65 (2018) |
| 4 | 5 | Kellar A, Tangtatco JA, Weinstein M, Saunders N: Subcutaneous Fat Necrosis of the Newborn With Initial Hypocalcemia and Familial Recurrence: A Case Report. J Cutan Med Surg 22: 223–225 (2018) |
| 5 | 6 | Kim SH: Subcutaneous Fat Necrosis of the Newborn: A Case Report. Korean J Radiol 78: 77 (2018) |
| 6 | 7 | Muzy G, Mayor SAS, Lellis RF: Subcutaneous fat necrosis of the newborn: clinical and histopathological correlation. An Bras Dermatol 93: 412–414 (2018) |
| 7 | 8 | Rato M, Catarino A, Claro C, Viana I: Cutaneous complication of perinatal hypoxia. Dermatol Online J 24 (2018) |
| 8 | 9 | Cheng JYW, Lee RSY: http://​www.hkjpaed.org​/​pdf/​2017;22;120-121.pdf (21.05.2018) |
| 9 | 10 | Di Bari A, Nead JA, Schurman SJ: Zoledronic acid for neonatal subcutaneous fat necrosis. Clin Case Rep 5: 567–569 (2017) |
| 10 | 11 | Garg A, Singhal R, Chaudhary S: Neonatal hypercalcemia secondary to subcutaneous fat necrosis presenting as severe dehydration.  Indian J Paediatr Dermatol 19: 146-147 (2017) |
| 11 | 12 | Khedr S, Piskorski A, Bingham AR, Goldstein J, Laptook AR, Paepe ME de: Occult Massive Visceral Fat Necrosis Following  Therapeutic Hypothermia for Neonatal Encephalopathy. Pediatr Dev Pathol 21: 1-5 (2018) |
| 12 | 13 | Kwon HS, Lee JH, Kim GM, Bae JM: Image Gallery: Subcutaneous fat necrosis of the newborn. Br J Dermatol 176: 32 (2017) |
| 13 | 14, 15, 16 ,17, 18 | Lara LG, Villa AV, Rivas MMO, Capella MS, Prada F, Enseñat MAG: Subcutaneous Fat Necrosis of the Newborn:  Report of Five Cases. Pediatr Neonatol 58: 85–88 (2017) |
| 14 | 19 | Onyiriuka AN, Utomi TE: Hypocalcemia Associated with Subcutaneous Fat Necrosis of the Newborn: Case Report and Literature  Review. Oman Med J 32: 518–521 (2017) |
| 15 | 20 | Patel NS, Carpenter TO, Genel M: Single dose of Bisphosphonate to treat infantile Hypercalcemia. AACE Clin Case Rep 3: e246-e250 (2017) |
| 16 | 21 | Sultana J, Yasmeen S, Nazme NI, Moslem MHM, Haque ZSM, Fatema NN: Subcutaneous Fat Necrosis of the Newborn: A Case Report.  J Bangladesh Coll Phys Surg 35: 192–195 (2017) |
| 17 | 22 | Chen K-L, Chen C-Y, Chiu H-C: Extensive subcutaneous fat necrosis after selective head cooling in a newborn with hypoxic ischemic encephalopathy. Dermatologica Sinica 34: 211–212 (2016) |
| 18 | 23 | Guzoglu N, Aksoy HT, Oguz SS, Fitoz S, Dilmen U: Growing Masses in a Preterm Newborn's Cheeks. Indian J Pediatr 83: 189–190 (2016) |
| 19 | 24 | Handa N, Kachhawa D, Soni JP, Jain VD, Rao P: Subcutaneous Fat Necrosis of the Newborn. Indian Journal of Neonatal Medicine  and Research 4 (2016) |
| 20 | 25 | Martínez de Zabarte Fernández JM, Laliena Aznar S, Corella Aznar E, Cuadrado Piqueras L, Oliván del Cacho MJ, Pinillos Pisón R: Necrosis grasa subcutánea con hipercalcemia persistente en un neonato tratado con hipotermia terapéutica. Reporte de un caso. Arch Argent Pediatr 114: e13-e16 (2016) |
| 21 | 26 | Quitadamo PA, Villani A, Cristalli PP, Marinelli M, Riganti A, Bisceglia M, Gatta A: Important hypercalcemia due to subcutaneous fat necrosis treated with pamidronate in an infant with severe hypoxic-ischemic encephalopathy. Journal of Pediatric and Neonatal Individualized Medicine 5: 1–5 (2016) |
| 22 | 27 | Schubert PT, Razak R, Jordaan HF: Fine-Needle Aspiration as a Method of Diagnosis of Subcutaneous Fat Necrosis of the Newborn. Pediatr Dermatol 33: e220–e221 (2016) |
| 23 | 28, 29 | Szpecht D, Bagnosz-Magnuszewska A, Szymankiewicz M, Gadzinowski J: Subcutaneous fat necrosis in neonates after  therapeutic hypothermia - report of two cases. Postepy Dermatol Alergol 33: 152–154 (2016) |
| 24 | 30 | Thomas JM, Bhandari J, Rytina E, Gass JK, Williams RM, Burrows NP: Subcutaneous Fat Necrosis of the Neonate with a Delayed  Second Eruption. Pediatr Dermatol 33: e134-136 (2016) |
| 25 | 31, 32 | Woo CH, Ham S, Lee H, Haw S, Choi M, Park H: http://​www.papersearch.net​/​thesis/​article.asp?key=3427644 (13.07.2018) |
| 26 | 33 | Anandan V, Rashmi S: Subcutaneous fat necrosis of newborn with asymptomatisc hypercalcemia. Stanley Medical Journal 2: 19–20 (2015) |
| 27 | 34 | Chikaodinaka AA, Jude AC: Subcutaneous Fat Necrosis of the Newborn: A Case Report of a Term Infant Presenting with Malaise and  Fever at Age of 9 Weeks. Case Rep Pediatr 2015: 1–5 (2015) |
| 28 | 35 | Gomes C, Lobo L, Azevedo AS, Simão C: Nefrocalcinose e Necrose Gorda do Tecido Celular Subcutâneo. Acta Med Port 28: 119–122 (2015) |
| 29 | 36, 37, 38, 39 | Grass B, Weibel L, Hagmann C, Brotschi B: Subcutaneous fat necrosis in neonates with hypoxic ischaemic encephalopathy registered in  the Swiss National Asphyxia and Cooling Register. BMC Pediatr 15: 73 (2015) |
| 30 | 40, 41 | Martins J, Maxaud A, Bah A-G, Prophette B, Maillard H, Bénéton N: Cytostéatonécrose après hypothermie thérapeutique contrôlée  chez deux nouveau-nés d'origine africaine. Arch Pediatr 22: 191–194 (2015) |
| 31 | 42, 43 | Messaoudi S, Seddiki AE, Chaalal M, Amrani R: La cytostéatonécrose du nouveau-né: à propos de deux observations. Pan Afr Med J 22:  34 (2015) |
| 32 | 44 | Mneimneh S, ELRajab O, Rajab M: Subcutaneous fat necrosis in newborn: case report. Int J Contemp Pediatrics 2: 164 (2015) |
| 33 | 45, 46 | Rubin G, Spagnut G, Morandi F, Valerio E, Cutrone M: Subcutaneous fat necrosis of the newborn. Clin Case Rep 3: 1017–1020 (2015) |
| 34 | 47 | Sahin S, Oncel MY, Alkan M, Bidev D, Oguz SS, Fitoz S, Dilmen U: Visceral fat necrosis in a newborn after whole body hypothermia.  JPediatr 166: 1545 (2015) |
| 35 | 48 | Tognetti L, Filippou G, Bertrando S, Picerno V, Buonocore G, Frediani B, Fimiani M, Rubegni P: Subcutaneous fat necrosis in a  newborn after brief therapeutic hypothermia: ultrasonographic examination. Pediatr Dermatol 32: 427–429 (2015) |
| 36 | 49 | Tuddenham E, Kumar A, Tarn A: Subcutaneous fat necrosis causing neonatal hypercalcaemia. BMJ Case Rep doi:10.1136/bcr-2014-  208460: 1–3 (2015) |
| 37 | 50 | Yagci-Küpeli B: Subcutaneous fat necrosis in a newborn after therapeutic hypothermia: A report of a case. J Clin Neonatol 4: 262–264  (2015) |
| 38 | 51 | Beuzeboc Gérard M, Aillet S, Bertheuil N, Delliere V, Thienot S, Watier E: Surgical management of subcutaneous fat necrosis of the  newborn required due to a lack of improvement: a very rare case. Br J Dermatol 171: 183–185 (2014) |
| 39 | 52 | Chacham S, Hassan S, Nagasravani J, Rao J, Reddy UN, Kumar AS: Subcutaneous Fat Necrosis in a Term Male Neonate with Perinatal  Asphyxia: A Case Reoprt. Sch J App Med Sci 2: 1014–1016 (2014) |
| 40 | 53 | De la Torre-Gutiérrez M, Padilla-Muñoz H, Pérez Rulfo-Ibarra D, Castillo-Villarruel F, Angulo-Castellanos E, Campos-Sierra A, Barrera-  Sánchez FJ, Stanley-Lucero MA, Alfaro-Castellanos DE, García-Magdaleno PE, Pérez-Gómez HR: Fat necrosis in a newborn. Case Report.  Revista Medica MD 5: 248–250 (2014) |
| 41 | 54 | Díaz Díaz J, Morante Valverde R, Delgado Muñoz MD, Matí Carreras E, Bustos Lozano G: Necrosis grasa subcutánea complicada tras  tratamiento con hipotermia terapéutica por encefalopatía hipóxico-isquémica grave. An Pediatr (Barc) 81: e36-e37 (2014) |
| 42 | 55, 56, 57, 58 | Jianping, Tang, Ye, Shu: Subcutaneous Fat Necrosis of the Newborn: Clinical Manifestations in Four Cases. Austin J Dermatolog 1:  1017 (2014) |
| 43 | 59 | Milankov O, Savić R, Bjelica M, Katanić D, Lovrenski J, Vučković N: Subcutaneous fat necrosis of the infant. Paediatr Croat 58: 231–234  (2014) |
| 44 | 60 | Niranjan U, Brooke N, Desai V, Natarajan A: Subcutaneous fat necrosis causing prolonged hypercalcemia in a neonate: An unusual case.  ESPE Abstracts (2014) 82 P-D-3-2-892, S.1 (2014) |
| 45 | 61 | Pérez Martínez E, Camprubí Camprubí M, Ramos Cebrián M, López AJ, Apodaca Saracho A, Lopez Ramos MG, García-Alix A: Treatment  with bisphosphonates in severe hypercalcemia due to subcutaneous fat necrosis in an infant with hypoxic-ischemic encephalopathy.  J Perinatol 34: 492–493 (2014) |
| 46 | 62 | Rahman A, Ameri AA, Shehri H, Mridha M: Subcutaneous Fat Necrois of Newborn (SCFN) Associated with Head Cooling in an Asphyxiated  Newborn - A case report. Bangladesh J Child Health 38: 101–103 (2014) |
| 47 | 63, 64 | Samedi VM, Yusuf K, Yee W, Obaid H, Al Awad EH: Neonatal hypercalcemia secondary to subcutaneous fat necrosis successfully treated  with pamidronate: a case series and literature review. AJP Rep 4: e93-e96 (2014) |
| 48 | 65, 66, 67, 68, 69, 70, 71 | Shumer DE, Thaker V, Taylor GA, Wassner AJ: Severe hypercalcaemia due to subcutaneous fat necrosis: presentation, management and  complications. Arch Dis Child Fetal Neonatal Ed 99: F419-421 (2014) |
| 49 | 72 | Sindhurakasturi, Patra LB, Sethi RK, Patra DP: A Case Report on Subcutaneous Fat Necrosis in Newborn. J of Evidence Based Med &  Hlthcare 1: 1329–1331 (2014) |
| 50 | 73 | Akcay A, Akar M, Oncel MY, Kızılelma A, Erdeve O, Oguz SS, Uras N, Dilmen U: Hypercalcemia due to subcutaneous fat necrosis in a  newborn after total body cooling. Pediatr Dermatol 30: 120–123 (2013) |
| 51 | 74 | Al Shidhani KS, Al Maani AS, Al Jabri AT: A Rare Presentation of a Newborn with Subcutaneous Fat Necrosis. Oman Med J 28: 1–3 (2013) |
| 52 | 75 | Calisici E, Oncel MY, Degirmencioglu H, Sandal G, Canpolat FE, Erdeve O, Oguz SS, Dilmen U: A neonate with subcutaneous fat necrosis after passive cooling: does polycythemia have an effect? Case Rep Pediatr 254089: 1–3 (2013) |
| 53 | 76 | Cevik G, Beken S, Aydin B, Dilli D, Zenciroglu A, Okumus N: Subcutaneous fat necrosis during hypothermia treatment in an asphyxiated  infant. Gaziantep Med J 19: 188–190 (2013) |
| 54 | 77 | Coondoo A, Lahiry R, Choudhury A, Sengupta S: Tender skin nodules in a newborn. Indian J Dermatol 58: 328 (2013) |
| 55 | 78, 79 | Gomes MPdCL, Porro AM, Enokihara MMSdS, Floriano MC: Subcutaneous fat necrosis of the newborn: clinical manifestations in two cases. An Bras Dermatol 88: 154–157 (2013) |
| 56 | 80 | Kuboi T, Kusaka T, Okazaki K, Kaku U, Kakinuma R, Kondo M, Nishida A: Subcutaneous fat necrosis after selective head cooling in an  infant. Pediatr Int 55: e23-e24 (2013) |
| 57 | 81 | Sfaihi L, Kmiha S, Aloulou H, Kamoun T, Hachicha M: Biphosphonate therapy in subcutaneous fat necrosis with hypercalcemia and  nephrocalcinosis in the newborn. J Neonatal Nurs 19: 76–79 (2013) |
| 58 | 82 | Tizki S, Lasry F, Elftoiki FZ, Hadj Khalifa H, Itri M, Khadir K, Benchikhi H: Intérêt de l'échographie rénale dans la cytostéatonécrose sous-  cutanée. Arch Pediatr 20: 768–771 (2013) |
| 59 | 83 | Tzvi-Behr S, Megged O, Schlesinger Y, Bin-Nun A, Sagi E, Becker-Cohen R: Subcutaneous fat necrosis. J Pediatr 163: 300 (2013) |
| 60 | 84 | Yaqub A, Khan EA, Anwar V: Neonatal subcutaneous fat necrosis. J Coll Physicians Surg Pak 23: 527–530 (2013) |
| 61 | 85 | Abilkassem R, Dini N, Oukabli M, Kmari M, Agadr A: Association of neonatal fat necrosis, hypertriglyceridemia and hypercalcemia: report of  an observation. Pan Afr Med J 11: 26 (2012) |
| 62 | 86 | Canpolat N, Özdil M, Kuruğoğlu S, Çalışkan S, Sever L: Nephrocalcinosis as a complication of subcutaneous fat necrosis of the newborn.  Turk J Pediatr 54: 667–670 (2012) |
| 63 | 87 | Hsiao F, Lamb P: https://​www.jaad.org​/​article/​S0190-9622(11)01491-5/​fulltext (31.07.2018) |
| 64 | 88 | Ghinescu CE, Kamalanathan AN, Morgan C: Unilateral radial nerve palsy in a newborn. Arch Dis Child Fetal Neonatal Ed 97: F153 (2012) |
| 65 | 89 | Haider S: Images in paediatrics: subcutaneous fat necrosis causing radial nerve palsy. BMJ Case Rep doi: 10.1136/bcr.10.2011.4904: 1–2 (2012) |
| 66 | 90, 91, 92 | Hogeling M, Meddles K, Berk DR, Bruckner AL, Shimotake TK, Cohen RS, Frieden IJ: Extensive subcutaneous fat necrosis of the newborn  associated with therapeutic hypothermia. Pediatr Dermatol 29: 59–63 (2012) |
| 67 | 93 | Savić DM, Stojanović ND, Stanković VD, Stojković AK, Čanović DS, Ninković SM, Milošević BZ, Cvetković AM: Subcutaneous fat necrosis  in newborns. Med Glas (Zenica) 9: 429–431 (2012) |
| 68 | 94 | Scheans P: Subcutaneous fat necrosis: a complication of neuroprotective cooling. Neonatal Netw 31: 409–412 (2012) |
| 69 | 95, 96 | Schubert PT, Razack R, Vermaak A, Jordaan HF: Fine-needle aspiration cytology of subcutaneous fat necrosis of the newborn: the cytology  spectrum with review of the literature. Diagn Cytopathol 40: 245–247 (2012) |
| 70 | 97 | Sivanandan S, Rabi Y, Kamaluddeen M, Akierman A, Lodha A: Subcutaneous fat necrosis as a complication of therapeutic hypothermia in  a term neonate. Indian J Pediatr 79: 664–666 (2012) |
| 71 | 98 | Woods AG, Cederholm CK: Subcutaneous fat necrosis and whole-body cooling therapy for neonatal encephalopathy. Adv Neonatal Care  12: 345–348 (2012) |
| 72 | 99 | Akin MA, Akin L, Coban D, Akcakus M, Balkanli S, Kurtoglu S: Post-operative subcutaneous fat necrosis in a newborn: a case report. Fetal  Pediatr Pathol 30: 363–369 (2011) |
| 73 | 100 | Akin MA, Akin L, Sarıcı D, Yılmaz I, Balkanlı S, Kurtoğlu S: Follow-up during early infancy of newborns diagnosed with subcutaneous fat  necrosis. J Clin Res Pediatr Endocrinol 3: 216–218 (2011) |
| 74 | 101 | Alaoui K, Abourazzak S, Oulmaati A, Hida M, Bouharrou A: An unusual complication of subcutaneous fat necrosis of the newborn. BMJ  Case Rep doi:10.1136/bcr.12.2010.3569: 1–3 (2011) |
| 75 | 102, 103, 104, 105, 106, 107, 108, 109, 110, 111, 112, 113, 114, 115, 116, 117, 118 | Alsubhi FS, Althunyan AM, Curtis CG, Clarke HM: Radial nerve palsy in the newborn: a case series. Canadian Medical Association journal  183: 1367–1370 (2011) |
| 76 | 119 | Gómez-Fernández C, Feito Rodríguez M, Collantes Bellido E, Ybarra Zabala M, Lucas Laguna R de: Placa indurada en la espalda de recién  nacida tras tratamiento con enfriamiento corporal total. An Pediatr (Barc) 74: 64–66 (2011) |
| 77 | 120 | Hakan N, Aydin M, Zenciroglu A, Demirel N, Okumus N, Cetinkaya S, Ipek MS: Alendronate for the treatment of hypercalcaemia due to neonatal subcutaneous fat necrosis. Eur J Pediatr 170: 1085-1087 (2011) |
| 78 | 121 | Landau Y, Berger I, Marom R, Mandel D, Ben Sira L, Fattal-Valevski A, Peylan T, Levi L, Dolberg S, Bassan H: Therapeutic hypothermia for  asphyxiated newborns: experience of an Israeli tertiary center. Isr Med Assoc J 13: 29–33 (2011) |
| 79 | 122 | Mitra S, Dove J, Somisetty SK: Subcutaneous fat necrosis in newborn-an unusual case and review of literature. Eur J Pediatr 170: 1107–1110 (2011) |
| 80 | 123, 124 | Oliveira ACS, Selores M, Pereira O: Fat necrosis of the newborn--report on two cases. An Bras Dermatol 86: 114-7 (2011) |
| 81 | 125 | Yuen NS, Ibrahim SBK: Picture of the month. Subcutaneous fat necrosis of the newborn. Arch Pediatr Adolesc Med 165: 563–564 (2011) |
| 82 | 126 | Marszałek A, Maciejewska J, Bowszyc-Dmochowska M, Prokurat A: Subcutaneous fat necrosis of the newborn - a case report and review of  literature. Pol J Pathol 61: 240–244 (2010) |
| 83 | 127 | Oza V, Treat J, Cook N, Tetzlaff MT, Yan A: Subcutaneous fat necrosis as a complication of whole-body cooling for birth asphyxia. Arch  Dermatol 146: 882–885 (2010) |
| 84 | 128 | Perrotta R, Virzì D, Tarico MS: A rare case of congenital ulcerated subcutaneous fat necrosis of the newborn. J Plast Reconstr Aesthet Surg  63: e801-e802 (2010) |
| 85 | 129 | Zifman E, Mouler M, Eliakim A, Nemet D, Pomeranz A: Subcutaneous fat necrosis and hypercalcemia following therapeutic hypothermia—a  patient report and review of the literature. J Pediatr Endocrinol Metab 23: 1185–1188 (2010) |
| 86 | 130 | Camp W, JR., Kress D, Jukic D: Subcutaneous fat necrosis of the newborn in association with meconium aspiration. J Am Acad Dermatol  60: AB148 (2009) |
| 87 | 131 | Kim JH, Jeong SY, Kim I-H, Son SW: Subcutaneous fat necrosis of the newborn associated with ventricular septal defect and patent ductus  arteriosus. Int J Dermatol 48: 1021–1023 (2009) |
| 88 | 132 | Ladoyanni E, Moss C, Brown RM, Ogboli M: Subcutaneous fat necrosis in a newborn associated with asymptomatic and uncomplicated  hypercalcemia. Pediatr Dermatol 26: 217–219 (2009) |
| 89 | 133 | Lombardi G, Cabano R, Bollani L, Del Forno C, Stronati M: Effectiveness of pamidronate in severe neonatal hypercalcemia caused by subcutaneous fat necrosis: a case report. Eur J Pediatr 168: 625–627 (2009) |
| 90 | 134 | Nair S, Nair SG, Borade A, Ramakrishnan K: Hypercalcemia and metastatic calcification in a neonate with subcutaneous fat necrosis. Indian  J Pediatr 76: 1155–1157 (2009) |
| 91 | 135 | Vasireddy S, Long SD, Sacheti B, Mayforth RD: MRI and US findings of subcutaneous fat necrosis of the newborn. Pediatr Radiol 39:  73–76 (2009) |
| 92 | 136 | Aljaser F, Weinstein M: A 1-week-old newborn with hypercalcemia and palpable nodules: subcutaneous fat necrosis. Canadian Medical  Association journal 178: 1653–1654 (2008) |
| 93 | 137 | Bonnemains L, Rouleau S, Sing G, Bouderlique C, Coutant R: Severe neonatal hypercalcemia caused by subcutaneous fat necrosis without any apparent cutaneous lesion. Eur J Pediatr 167: 1459–1461 (2008) |
| 94 | 138 | Farinelli P, Gattoni M, Delrosso G, Boggio P, Raselli B, Merlo E, Valente G, Colombo E: Eosinophilic granules in subcutaneous fat necrosis  of the newborn: what do they mean? J Cutan Pathol 35: 1073–1074 (2008) |
| 95 | 139 | Ghergherehchi R: Complication of Subcutaneous Fat Necrosis of the Newborn: A Case Report and Review of the Literature. Res J Biol Sci  3: 1004–1007 (2008) |
| 96 | 140 | Karimi A, Sayyahfar S., Jadali F, Fahimzad A, Armin S, Ghorubi J, Jahromi MH, Saket S: Subcutaneous fat necrosis of the newborn  complicated with hypercalcemia. Pak J Med Sci 24: 178–180 (2008) |
| 97 | 141 | Monica JT, Waters PM, Bae DS: Radial nerve palsy in the newborn: a report of four cases and literature review. J Pediatr Orthop 28:  460–462 (2008) |
| 98 | 142 | Raimer L, McCarthy RA, Raimer D, Colome-Grimmer M: Congenital Volkmann ischemic contracture: a case report. Pediatr Dermatol 25:  352–354 (2008) |
| 99 | 143 | Yu JE, Pai K-S, Park MS: Congenital cavernous hemangioma exhibiting subcutaneous fat necrosis. Clin Pediatr (Phila) 47: 74–76 (2008) |
| 100 | 144 | Aucharaz KS, Baker EL, Millman GC, Ball RJ: Neonatal subcutaneous fat necrosis with characteristic rash and hypercalcaemia. Arch Dis  Child Fetal Neonatal Ed 92: F304 (2007) |
| 101 | 145 | Germanaud D, Hadj-Rabia S, Parsy C, Abadie V: Cytostéatonécrose néonatale compliquée d'une hypercalcémie symptomatique: efficacité  des corticoïdes à faible dose. Arch Pediatr 14: 167–169 (2007) |
| 102 | 146 | Isaiah JH, Chan AKJ: Subcutaneous fat necrosis of the newborn and lactic acidosis. Pediatr Dermatol 24: 435–436 (2007) |
| 103 | 147, 148, 149, 150, 151, 152, 153, 154, 156, 157, 158, 159, 160, 161, 162 | Mahé E, Girszyn N, Hadj-Rabia S, Bodemer C, Hamel-Teillac D, Prost Y de: Subcutaneous fat necrosis of the newborn: a systematic evaluation of risk factors, clinical manifestations, complications and outcome of 16 children. Br J Dermatol 156: 709–715 (2007) |
| 104 | 163 | Pai SA, Nagesh K, Radhakrishnan CN: Subcutaneous fat necrosis of the newborn mimicking generalized lymphadenopathy. Indian J  Dermatol Venereol Leprol 73: 357–358 (2007) |
| 105 | 164, 165, 166, 167, 168, 1691 170 | Singalavanija S, Limponsanurak W, Wannaprasert T: Subcutaneous fat necrosis of the newborn. J Med Assoc Thai 90: 1214–1220 (2007) |
| 106 | 171 | Tajirian A, Ross R, Zeikus P, Robinson-Bostom L: Subcutaneous fat necrosis of the newborn with eosinophilic granules. J Cutan Pathol 34:  588–590 (2007) |
| 107 | 172 | Trullemans B, Bottu J, van Nieuwenhuyse J-P: Etidronate per os dans le cadre d'une hypercalcémie secondaire à une cytostéatonécrose  compliquée de néphrocalcinose. Arch Pediatr 14: 170–172 (2007) |
| 108 | 173 | Zaulyanov LL, Jacob SE, Elgart GW, Schachner L: Subcutaneous fat necrosis of the newborn and hyperferritinemia. Pediatr Dermatol 24:  93 (2007) |
| 109 | 174, 175, 176, 177 | Alos N, Eugène D, Fillion M, Powell J, Kokta V, Chabot G: Pamidronate: Treatment for severe hypercalcemia in neonatal subcutaneous fat  necrosis. Horm Res Paediatr 65: 289–294 (2006) |
| 110 | 178 | Borgia F, Pasquale L de, Cacace C, Meo P, Guarneri C, Cannavo SP: Subcutaneous fat necrosis of the newborn: be aware of  hypercalcaemia. J Paediatr Child Health 42: 316–318 (2006) |
| 111 | 179 | Diamantis S, Bastek T, Groben P, Morrell D: Subcutaneous fat necrosis in a newborn following icebag application for treatment of supraventricular tachycardia. J Perinatol 26: 518–520 (2006) |
| 112 | 180 | Karochristou K, Siahanidou T, Kakourou-Tsivitanidou T, Stefanaki K, Mandyla H: Subcutaneous fat necrosis associated with severe  hypocalcaemia in a neonate. J Perinatol 26: 64–66 (2006) |
| 113 | 181 | Mesquita M, Matsuda N, Siqueira S, Santos H: Subcutaneous fat necrosis in a newborn. Einstein (Sao Paulo) 4: 22–24 (2006) |
| 114 | 182 | Srinath G, Cohen M: Imaging findings in subcutaneous fat necrosis in a newborn. Pediatr Radiol 36: 361–363 (2006) |
| 115 | 183 | Vijayakumar M, Prahlad N, Nammalwar BR, Shanmughasundharam R: Subcutaneous fat necrosis with hypercalcemia. Indian Pediatr 43:  360–363 (2006) |
| 116 | 184 | Acun C, Kargi E, Ustundag G, Hosnuter M, Numanoglu G: Subcutaneous fat necrosis of the newborn. Plast Reconstr Surg 115: 1790–1792  (2005) |
| 117 | 185 | Parvathidevi GK, Vijayashankar MR, Belagavi CS, Deepak, Vijaya, Narendra G, Ramesh: Cytological diagnosis of subcutaneous fat  necrosis of newborn: a case report. Dermatol Online J 11: 20 (2005) |
| 118 | 186 | Shirin M, Mamun MAA, Hossain MM: Subcutaneous fat necrosis. Dhaka Shishu (Children) Hospital journal 21: 78–80 (2005) |
| 119 | 187, 188 | Fenniche S, Daoud L, Benmously R, Ben Ammar F, Khelifa I, Chaabane S, Mokhtar I: Subcutaneous fat necrosis: report of two cases.  Dermatol Online J 10: 12 (2004) |
| 120 | 189, 190 | Miller SF: Resolution of calcific brown fat necrosis associated with prostaglandin therapy for cyanotic congenital heart disease in neonates:  report of two cases. Pediatr Radiol 34: 919–923 (2004) |
| 121 | 191 | Poblete-Gutiérrez P, Ott H, Krischer S, Grussendorf-Conen E-I, Frank J: Adiponecrosis subcutanea neonatorum bei konnataler Pneumonie.  Hautarzt 55: 67–70 (2004) |
| 122 | 192 | Barbier C, Cneude F, Deliège R, El Kohen R, Kremy O, Leclerc F: Cytostéatonécrose néonatale: attention à l'hypercalcémie sévère. Arch  Pediatr 10: 713–715 (2003) |
| 123 | 193 | Dudink J, Walther FJ, Beekman RP: Subcutaneous fat necrosis of the newborn: hypercalcaemia with hepatic and atrial myocardial  calcification. Arch Dis Child Fetal Neonatal Ed 88: F343-345 (2003) |
| 124 | 194 | Fernando RA, Somers S, Edmonson RD, Sidhu PS: https://​www.neonet.ch​/​files/​8514/​2591/​5178/​February_2003.pdf (21.05.2018) |
| 125 | 195 | Haileamlak A: Hypercalcemia in association with subcutaneous fat necrosis of the newborn. Ethiop J Health Sci 13: 77–80 (2003) |
| 126 | 196 | Hung S-H, Tsai W-Y, Tsao P-N, Chou H-C, Hsieh W-S: Oral clodronate therapy for hypercalcemia related to extensive subcutaneous fat necrosis in a newborn. J Formos Med Assoc 102: 801–804 (2003) |
| 127 | 197 | Noorollah CDO, Choudhury SDO, Rozenberg SDO: A Case of Subcutaneous Fat Necrosis of the Newborn. Journal of the American  Osteopathic College of Dermatology 27: 32–33 (2003) |
| 128 | 198 | Tran JT, Sheth AP: Complications of subcutaneous fat necrosis of the newborn: a case report and review of the literature. Pediatr Dermatol  20: 257–261 (2003) |
| 129 | 199 | Balfour E, Antaya RJ, Lazova R: Subcutaneous fat necrosis of the newborn presenting as a large plaque with lobulated cystic areas. Cutis  70: 169–173 (2002) |
| 130 | 200 | Bellini C, Oddone M, Biscaldi E, Serra G: Radiological case of the month. Subcutaneous fat necrosis of the newborn. Arch Pediatr Adolesc  Med 155: 1381–1382 (2001) |
| 131 | 201 | Herman TE, Siegel MJ: Special imaging casebook. Hypoplastic left heart, prostaglandin therapy gastric focal foveolar hyperplasia and  brown-fat necrosis. J Perinatol 21: 263–265 (2001) |
| 132 | 202 | Khan N, Licata A, Rogers D: Intravenous bisphosphonate for hypercalcemia accompanying subcutaneous fat necrosis: a novel treatment  approach. Clin Pediatr (Phila) 40: 217–219 (2001) |
| 133 | 203 | Lee SK, Lee JH, Han CH, Ahn YM, Choi YS, Kim IO: Calcified subcutaneous fat necrosis induced by prolonged exposure to cold weather:  a case report. Pediatr Radiol 31: 294–295 (2001) |
| 134 | 204 | Wiadrowski TP, Marshman G: Subcutaneous fat necrosis of the newborn following hypothermia and complicated by pain and  hypercalcaemia. Australas J Dermatol 42: 207–210 (2001) |
| 135 | 205 | Narchi H, Chellapa C: Unusual location of subcutaneous fat necrosis in a term newborn. Clin Pediatr (Phila) 39: 254 (2000) |
| 136 | 206 | Anderson DR, Narla LD, Dunn NL: Subcutaneous fat necrosis of the newborn. Pediatr Radiol 29: 794–796 (1999) |
| 137 | 207, 208, 209, 210, 211, 212, 213, 214, 215, 216, 217 | Burden AD, Krafchik BR: Subcutaneous fat necrosis of the newborn: a review of 11 cases. Pediatr Dermatol 16: 384–387 (1999) |
| 138 | 218 | Ghirri P, Bottone U, Coccoli L, Bernardini M, Vuerich M, Cuttano A, Riparbelli C, Pellegrinetti G, Boldrini A: Symptomatic hypercalcemia in  the first months of life: calcium-regulating hormones and treatment. J Endocrinol Invest 22: 349–353 (1999) |
| 139 | 219, 220, 221 | Hayman M, Roland EH, Hill A: Newborn radial nerve palsy: report of four cases and review of published reports. Pediatr Neurol 21: 648–651  (1999) |
| 140 | 222, 223, 224 | Lum CK, Solomon IL, Bachrach LK: Asymptomatic hypercalcemia in subcutaneous fat necrosis. Clin Pediatr (Phila) 38: 547–550 (1999) |
| 141 | 225 | Raboi CA, Smith W: Brown fat necrosis in the setting of congenital heart disease and prostaglandin E1 use: a case report. Pediatr Radiol 29:  61–63 (1999) |
| 142 | 226 | Repiso-Jiménez JB, Márquez J, Sotillo I, García-Bravo B, Camacho F: Subcutaneous fat necrosis of the newborn. J Eur Acad Dermatol  Venereol 12: 254–257 (1999) |
| 143 | 227 | Rice AM, Rivkees SA: Etidronate therapy for hypercalcemia in subcutaneous fat necrosis of the newborn. J Pediatr 134: 349–351 (1999) |
| 144 | 228 | Varan B, Gürakan B, Ozbek N, Emir S: Subcutaneous fat necrosis of the newborn associated with anemia. Pediatr Dermatol 16: 381–383  (1999) |
| 145 | 229 | Craig JE, Scholz TA, Vanderhooft SL, Etheridge SP: Fat necrosis after ice application for supraventricular tachycardia termination. J Pediatr  133: 727 (1998) |
| 146 | 230 | Hernández-Martín A, Unamuno P de, Fernández-López E: Congenital ulcerated subcutaneous fat necrosis of the newborn. Dermatology  197: 261–263 (1998) |
| 147 | 231 | Rosbotham JL, Johnson A, Haque KN, Holden CA: Painful subcutaneous fat necrosis of the newborn associated with intra-partum use of a  calcium channel blocker. Clin Exp Dermatol 23: 19–21 (1998) |
| 148 | 232 | Scales JW, Krowchuk DP, Schwartz RP, Jorizzo JL: An infant with firm, fixed plaques. Arch Dermatol 134: 425–426 (1998) |
| 149 | 233 | Mather MK, Sperling LC, Sau P: Subcutaneous fat necrosis of the newborn. Int J Dermatol 36: 450–452 (1997) |
| 150 | 234, 235 | Norton KI, Som PM, Shugar JM, Rothchild MA, Popper L: Subcutaneous fat necrosis of the newborn: CT findings of head and neck  involvement. AJNR Am J Neuroradiol 18: 547–550 (1997) |
| 151 | 236, 237, 238, 320 | Chuang SD, Chiu HC, Chang CC: Subcutaneous fat necrosis of the newborn complicating hypothermic cardiac surgery. Br J Dermatol 132:  805–810 (1995) |
| 152 | 239, 240 | Gu LL, Daneman A, Binet A, Kooh SW: Nephrocalcinosis and nephrolithiasis due to subcutaneous fat necrosis with hypercalcemia in two  full-term asphyxiated neonates: sonographic findings. Pediatr Dermatol 25: 142–144 (1995) |
| 153 | 241, 242 | Sharata H, Postellon DC, Hashimoto K: Subcutaneous fat necrosis, hypercalcemia, and prostaglandin E. Pediatr Dermatol 12: 43–47 (1995) |
| 154 | 243 | Cabral JE, Ayres SdS, Ferreira LC, Albertoni DR: Necrose gordurosa do subcutâneo ou adipo necrose do recém-nascido. Descrição de um  caso. J Pediatr (Rio J) 70: 178–180 (1994) |
| 155 | 244 | Carraccio C, Papadimitriou J, Feinberg P: Subcutaneous fat necrosis of the newborn: link to maternal use of cocaine during pregnancy. Clin  Pediatr (Phila) 33: 317–318 (1994) |
| 156 | 245 | Darmstadt GL, Kanzler MH: Pathological case of the month. Subcutaneous fat necrosis of the newborn. Arch Pediatr Adolesc Med 148: 61–62 (1994) |
| 157 | 246 | Lewis HM, Ferryman S, Gatrad AR, Moss C: Subcutaneous fat necrosis of the newborn associated with hypercalcaemia. J R Soc Med 87:  482–483 (1994) |
| 158 | 247 | Hicks MJ, Levy ML, Alexander J, Flaitz CM: Subcutaneous fat necrosis of the newborn and hypercalcemia: case report and review of the  literature. Pediatr Dermatol 10: 271–276 (1993) |
| 159 | 248 | Higgins JN, Haddock JA, Shaw DG: Case report: soft tissue and perivisceral calcification occurring in an infant: a case of brown fat necrosis.  Br J Radiol 66: 366–368 (1993) |
| 160 | 249 | Janssens PM, Vonk J, Demacker PN: Hypertriglyceridaemia in a case of subcutaneous fat necrosis in a newborn. Ann Clin Biochem 30:  482–484 (1993) |
| 161 | 250, 251 | Kruse K, Irle U, Uhlig R: Elevated 1,25-dihydroxyvitamin D serum concentrations in infants with subcutaneous fat necrosis. J Pediatr 122:  460–463 (1993) |
| 162 | 252 | Rombouts JJ, Debauche C, Verellen G, Lyon G: Paralysies congénitales par compression. A propos de quatre observations. Ann Chir Main  Memb Super 12: 39–44 (1993) |
| 163 | 253 | Walker WP, Smith RJ, Cohen MB: Fine-needle aspiration biopsy of subcutaneous fat necrosis of the newborn. Diagn Cytopathol 9: 329–332  (1993) |
| 164 | 254 | Herland CC, Holden CA: Subcutaneous Fat necrosis of the newborn. Br J Dermatol 127: 82–84 (1992) |
| 165 | 255 | Lewis A, Cowen P, Rodda C, Dyall-Smith D: Subcutaneous fat necrosis of the newborn complicated by hypercalcaemia and  thrombocytopenia. Australas J Dermatol 33: 141–144 (1992) |
| 166 | 256, 257 | Glover MT, Catterall MD, Atherton DJ: Subcutaneous fat necrosis in two infants after hypothermic cardiac surgery. Pediatr Dermatol 8:  210–212 (1991) |
| 167 | 258 | Fernández-López E, Garcia-Dorado J, Unamuno P de, Heras I, Santos-Borbujo J, Armijo M: Subcutaneous fat necrosis of the newborn and  idiopathic hypercalcemia. Dermatologica 180: 250–254 (1990) |
| 168 | 259 | Jardine D, Atherton DJ, Trompeter RS: Sclerema neonatorum and subcutaneous fat necrosis of the newborn in the same infant. Eur J  Pediatr 150: 125–126 (1990) |
| 169 | 260 | Wolach B, Raas-Rothschild A, Vogel R, Choc L, Metzker A: Subcutaneous fat necrosis with thrombocytopenia in a newborn infant.  Dermatologica 181: 54–55 (1990) |
| 170 | 261 | Friedman SJ, Winkelmann RK: Subcutaneous fat necrosis of the newborn: light, ultrastructural and histochemical microscopic studies.  J Cutan Pathol 16: 99–105 (1989) |
| 171 | 262 | Finne PH, Sanderud J, Aksnes L, Bratlid D, Aarskog D: Hypercalcemia with increased and unregulated 1,25-dihydroxyvitamin D production  in a neonate with subcutaneous fat necrosis. J Pediatr 112: 792–794 (1988) |
| 172 | 263 | Lusk RP, Greiman MC: Subcutaneous fat necrosis in infancy. Otolaryngol Head Neck Surg 99: 520–523 (1988) |
| 173 | 264 | Balázs M: Subcutaneous fat necrosis of the newborn with emphasis on ultrastructural studies. Int J Dermatol 26: 227–230 (1987) |
| 174 | 265, 266, 267, 268, 269 | Larralde de Luna M, Cicioni V: Subcutaneous fat necrosis in the Newborn. Revista Argentina de Dermatologia 68: 18–24 (1987) |
| 175 | 270 | Norwood-Galloway A, Lebwohl M, Phelps RG, Raucher H: Subcutaneous fat necrosis of the newborn with hypercalcemia. J Am Acad  Dermatol 16: 435–439 (1987) |
| 176 | 271 | Silverman AK, Michels EH, Rasmussen JE: Subcutaneous fat necrosis in an infant, occurring after hypothermic cardiac surgery. Case report  and analysis of etiologic factors. J Am Acad Dermatol 15: 331–336 (1986) |
| 177 | 272 | Yasuda T, Sunami S, Ogura N, Nishioka T, Nakajima H: Infantile hypercalcemia with subcutaneous fat necrosis. Report of a case with  studies on the pathogenesis of hypercalcemia. Acta Paediatr Scand 75: 1042–1045 (1986) |
| 178 | 273 | Katz DA, Huerter C, Bogard P, Braddock SW: Subcutaneous fat necrosis of the newborn. Arch Dermatol 120: 1517–1518 (1984) |
| 179 | 274 | Moreno-Gimenez JC, Hernández-Aguado I, Arguisjuela MT, Camacho-Martinez F: Subcutaneous-fat necrosis of the newborn. J Cutan  Pathol 10: 277–280 (1983) |
| 180 | 275 | Mogilner BM, Alkalay A, Nissim F, Frumkin A: Subcutaneous fat necrosis of the newborn. Clin Pediatr (Phila) 20: 748–750 (1981) |
| 181 | 276 | Thomsen RJ: Subcutaneous fat necrosis of the newborn and idiopathic hypercalcemia. Report of a case. Arch Dermatol 116: 1155–1158  (1980) |
| 182 | 277 | Dawson TA, Slattery C: Subcutaneous fat necrosis of the newborn and acute pancreatitis. Br J Dermatol 101: 359 (1979) |
| 183 | 278, 279 | Veldhuis JD, Kulin HE, Demers LM, Lambert PW: Infantile hypercalcemia with subcutaneous fat necrosis: endocrine studies. J Pediatr 95:  460–462 (1979) |
| 184 | 280 | Oswalt GC, Montes LF, Cassady G: Subcutaneous fat necrosis of the newborn. J Cutan Pathol 5: 193–199 (1978) |
| 185 | 281, 282, 283, 284 | Pasyk K: Studies on subcutaneous fat necrosis of the newborn. Virchows Arch A Pathol Anat Histol 379: 243–259 (1978) |
| 186 | 285 | Williams JL, Capitanio MA, Harcke HT: Bone scanning in neonatal subcutaneous fat necrosis. J Nucl Med 19: 861–863 (1978) |
| 187 | 286, 287 | Tsuji T: Subcutaneous fat necrosis of the newborn: Light and electron microscopic studies. Br J Dermatol 95: 407–416 (1976) |
| 188 | 288, 289 | Sharlin DN, Koblenzer P: Necrosis of subcutaneous fat with hypercalcemia. A puzzling and multifaceted disease. Clin Pediatr (Phila) 9: 290–294 (1970) |
| 189 | 290, 291 | Duhn R, Schoen EJ, Siu M: Subcutaneous fat necrosis with extensive calcification after hypothermia in two newborn infants. Pediatrics 41:  661–664 (1968) |
| 190 | 292 | Kropp H: Fieberhafter Verlauf bei Adiponekrosis subcutanea neonatorum mit schweren allgemeinen Krankheitserscheinungen.  Z Kinderheilkd 102: 289–296 (1968) |
| 191 | 293 | Truckenbrodt H, Antener I, Lampert F: Hypercalcämie und Adiponecrosis subcutanea mit Kalkablagerungen im frühen Säuglingsalter.  Z Kinderheilkd 95: 61–73 (1966) |
| 192 | 294, 295, 296, 297, 298 | Weary PE, Graham GF, Selden RF: Subcutaneous fat necrosis of the newborn. South Med J 59: 960–965 (1966) |
| 193 | 299 | McAleer JK, Mercer RD: Subcutaneous fat necrosis with calcifications and hypercalcemia in an infant. Report of a case. Cleve Clin Q 31:  179–183 (1964) |
| 194 | 300 | Wilkerson JA: Idiopathic infantile hypercalcemia, with subcutaneous fat necrosis. Am J Clin Pathol 41: 390–401 (1964) |
| 195 | 301 | Barltrop D: Hypercalcemia associated with neonatal subcutaneous fat necrosis. Arch Dis Child 38: 516–518 (1963) |
| 196 | 302, 303 | Browne KW, Burman D: Subcutaneous fat necrosis of the newborn. J Obstet Gynaecol Br Emp 65: 819–822 (1958) |
| 197 | 304 | Martin MM, Steven EM: Subcutaneous fat necrosis of the newborn with calcification of the tissues. Arch Dis Child 32: 146–148 (1957) |
| 198 | 305 | Clay P: November 25, 1955 Cases. Proc R Soc Med 49: 595–603 (1956) |
| 199 | 306 | Blake HA, Goyette EM, Lyter CS, Swan H: Subcutaneous fat necrosis complicating hypothermia. J Pediatr 46: 78–80 (1955) |
| 200 | 307 | Belisario JC: Report of a case of subcutaneous fat necrosis of the newborn (? sclerema neonatorum) with a brief review of some relevant literature. Aust J Dermatol 2: 94–98 (1953) |
| 201 | 308 | Collins HA, Stahlman M, Scott HW: The occurrence of subcutaneous fat necrosis in an infant following induced hypothermia used as an  adjuvant in cardic surgery. Ann Surg 138: 880–885 (1953) |
| 202 | 309 | Ivy RE, Howard FH: Subcutaneous fat necrosis of the newborn infant; report of a case in an infant born by cesarean section, and with no  anoxia. J Pediatr 42: 600–602 (1953) |
| 203 | 310, 311, 312 | Holzel A: Subcutaneous fat necrosis of the newborn. Arch Dis Child 26: 89–91 (1951) |
| 204 | 313 | Lightwood R: Radial nerve palsy associated with localized subcutaneous fat necrosis in the newborn. Arch Dis Child 26: 436–437 (1951) |
| 205 | 314 | Noojin RO, Pace BF, Davis HG: Subcutaneous fat necrosis of the newborn; certain etiologic considerations. J Invest Dermatol 12: 331–334  (1949) |
| 206 | 315, 316, 317, 318, 319, 320 | Fox H: Subcutaneous fat necrosis of the New-born. AMA Arch Derm Syphilol 27: 237 (1933) |

**Table S2:** Excluded references and reason for exclusion

|  | **Reference** | **Reason for exclusion** |
| --- | --- | --- |
| 207 | Adamama-Moraitou KK, Prassinos NN, Galatos AD, Tontis DK, Rallis TS: Isolated abdominal fat tissue inflammation and  necrosis in a cat. J Feline Med Surg 10: 192–197 (2008) | Animal study |
| 208 | Agarwal N, Pitchumoni CS: Acute pancreatitis: a multisystem disease. Gastroenterologist 1: 115–128 (1993) | Other underlying disease: pancreatitis |
| 209 | Al-Maawali AA, Miller E, Schulze A, Yoon G, Blaser SI: Subcutaneous fat pads on body MRI – an early sign of congenital  disorder of glycosylation PMM2-CDG (CDG1a). Pediatr Radiol 44: 222–225 (2014) | Other underlying disease: necrotizing fasciitis |
| 210 | Alos N, Chabot G: Importance of the renal calcium load as putative predictor for nephrocalcinosis in subcutaneous fat  necrosis associated with severe hypercalcemia. Horm Res Paediatr 70: 256 (2008) | No case report |
| 211 | Anbari R: Subcutaneous fat necrosis of the newborn (Adiponecrosis subcutanea neonatorum). Q Rev Pediatr 16: 206–  207 (1961) | No case report |
| 212 | Andersen UM: Adiponecrosis subcutanea neonatorum. Ugeskr Laeger 157: 2725–2726 (1995) | No case report |
| 213 | Appell RG, Nützenadel W, Schütze U: Die nekrotisierende Fasciitis beim Neugeborenen. Z Kinderchir 32: 175–178 (1981) | Other underlying disease |
| 214 | Assoc E: https://​www.dermnetnz.org​/​topics/​subcutaneous-fat-necrosis-of-the-newborn-pathology?utm_source=TrendMD&utm_medium=cpc&utm_campaign=DermNet_NZ_TrendMD_0 (21.05.2018) | No case report |
| 215 | Assor E, Grasemann C, Parker KT, Sochett EB: Infantile Hypercalcemia: Relationship of Dietary Intake of Calcium and  Vitamin D to Serum and Urine Levels. 30th Annual Meeting of the American-Society-for-Bone-and-Mineral-Research,  Montreal, CANADA; September 12 -16, 2008. J Bone Miner Res 23: 369 (2008) | No case report |
| 216 | Aucharaz KS, Baker EL, Millman GC, Ball RJ: Treatment of hypercalcaemia in subcutaneous fat necrosis is  controversial. Horm Res Paediatr 68: 31 (2007) | Duplicate case report |
| 217 | Ayeche R, Ben Radhia F, Chabchoub A, Jebnoun S, Siala-Gaigi S, Khrouf N: La cytostéatonécrose generalisée chez le  nouveau-né. A propos de trois observations. Tunis Med 72: 450–455 (1994) | No access to the full text possible, no exact case information in the abstract |
| 218 | Azzopardi D, Robertson NJ, Bainbridge A, Cady E, Charles-Edwards G, Deierl A, Fagiolo G, Franks NP, Griffiths J,  Hajnal J, Juszczak E, Kapetanakis B, Linsell L, Maze M, Omar O, Strohm B, Tusor N, Edwards AD: Moderate  hypothermia within 6 h of birth plus inhaled xenon versus moderate hypothermia alone after birth asphyxia (TOBY-Xe):  a proof-of-concept, open-label, randomised controlled trial. Lancet Neurol 15: 145–153 (2016) | No case report |
| 219 | Bachrach LK, Lum CK: Etidronate in subcutaneous fat necrosis of the newborn. J Pediatr 135: 530–531 (1999) | No case report |
| 220 | Balázs M, Csermely A, Boros V: Adiponecrosis subcutanea neonatorum. Orv Hetil 127: 339–341 (1986) | Other language except English, French, Spanish, Italian. No Abstract available in English |
| 221 | Barsukov VS: Morfologichekie aspekty nesvoevremennogo zhirogo metamorfoza vilochkovoĭ zhelezy. Ark Patol 49:  44–51 (1987) | Other language except English, French, Spanish, Italian. No Abstract available in English |
| 222 | Basu S, Terrett L, Alvi S: https://​www.endocrine-abstracts.org​/​ea/​0030/​ea0030p13 (13.07.2018) | Other primary disease: hypoparathyroidism |
| 223 | Bégon E, Blum L, Petitjean B, Jacomet L, Merbouche S, Moguelet P, Bachmeyer C: Adiponécrose sous-cutanée du  nouveau-né (cytostéatonécrose) et hypercalcémie après hypothermie thérapeutique. Ann Dermatol Venereol 139:  601–602 (2012) | No access to the full text possible, no exact case information in the abstract |
| 224 | Beneggi A, Adamoli P, Conforto F, Bonora G: Adiponecrosi del neonato. Pediatr Med Chir 17: 281–282 (1995) | No case report |
| 225 | Bergstein KR, Jacobsen RB, Jacobsen BB, Christesen HT: Effektiv behandling af hyperkalcaemi ved neonatal subkutan  fedtnekrose. Ugeskr Laeger 172: 2096–2097 (2010) | No access to the full text possible, no exact case information in the abstract |
| 226 | Bethenod M, Bourrelier V, Brun M: Un cas de cyto-stéatonécrose sous-cutanée du nouveau-né. Pediatrie 19: 995–998 (1964) | Other primary disease |
| 227 | Bikle DD, Patzek S, Wang Y: Physiologic and pathophysiologic roles of extra renal CYP27b1: Case report and review.  Bone Rep 8: 255–267 (2018) | No case report |
| 228 | Bona G, Barberis L, Zaffaroni M, Bundino S, Zina A: Adiponecrosi sottocutanea del neonato. Minerva Pediatr 37: 679–  683 (1985) | No case report |
| 229 | Bosler DS, Amin MB, Gulli F, Malhotra RK: Unusual case of calciphylaxis associated with metastatic breast carcinoma.  Am J Dermatopathol 29: 400–403 (2007) | Other primary disease: breast cancer |
| 230 | Cabrera HN, Rodríguez A, Savoia J, Costa JA: Necrosis grasa subcutánea del recién nacido. A propósito de dos casos. Med Cutan Ibero Lat Am 11: 11–16 (1983) | No access to the full text possible |
| 231 | Caluwaerts S, Lambin S, van Bree R, Peeters H, Vergote I, Verhaeghe J: Diet-induced obesity in gravid rats engenders  early hyperadiposity in the offspring. Metabolism 56: 1431–1438 (2007) | Animal study |
| 232 | Cameselle D, Islas D, Montenegro T, Afonso JL, Hernández B: Nódulos subcutáneos en un recién nacido. Actas Dermosifiliogr 97: 218–220 (2006) | No access to the full text possible |
| 233 | Caple JI, Reyes S: Subcutaneous fat necrosis of the newborn: a case presentation. J Perinatol 16: 140–141 (1996) | No access to the full text possible |
| 234 | Cham PMH, Drolet BA, Segura AD, Esterly NB: Congenital Volkmann ischaemic contracture: a case report and review.  Br J Dermatol 150: 357–363 (2004) | Other underlying disease: neonatal compartment syndrome |
| 235 | Chen J-S, Liu W-C, Yang KC, Chen L-W, Huang J-S, Chang H-T: Reconstruction with bilateral pedicled TRAM flap for  paraffinoma breast. Plast Reconstr Surg 115: 96–104 (2005) | Other underlying disease: breast cancer |
| 236 | Chen TH, Shewmake SW, Hansen DD, Lacey HL: Subcutaneous fat necrosis of the newborn. A case report. Arch  Dermatol 117: 36–37 (1981) | No access to the full text possible |
| 237 | Choi SY, Choi HY, Myung KB, Park EA: A Case of Subcutaneous Fat Necrosis of the Newborn. Ewha Medical Journal  19: 73–78 (1996) | Other language except English, French, Spanish, Italian. No Abstract available in English |
| 238 | Cook JS, Stone MS, Hansen JR: Hypercalcemia in association with subcutaneous fat necrosis of the newborn: studies  of calcium-regulating hormones. Pediatrics 90: 93–96 (1992) | No case report |
| 239 | Cooper A, Betts JM, Pereira GR, Ziegler MM: Taurine deficiency in the severe hepatic dysfunction complicating total  parenteral nutrition. J Pediatr Surg 19: 462–466 (1984) | Other underlying disease: dysfunction of the liver |
| 240 | Courteau C, Samman K, Ali N, Riley P, Wintermark P: Macrosomia and haemodynamic instability may represent risk  factors for subcutaneous fat necrosis in asphyxiated newborns treated with hypothermia. Acta Paediatr 105: 396-405  (2016) | No case report |
| 241 | Csermely A, Balázs M: Elektronmikroszkópos vizsgálatok újszülöttkori subcutan adiponecrosisban. Morphol Igazsagugyi  Orv Sz 26: 181–186 (1986) | No case report |
| 242 | Cunningham K, Atkinson SA, Paes BA: Subcutaneous fat necrosis with hypercalcemia. Can Assoc Radiol J 41: 158–159  (1990) | No access to the full text possible |
| 243 | Cunningham K, Paes BA: Subcutaneous fat necrosis of the newborn with hypercalcemia: a review. Neonatal Netw 10: 7–14 (1991) | No case report |
| 244 | Dasgupta A, Ghosh RN, Pal RK, Mukherjee N: Sclerema neonatorum--histopathologic study. Indian J Pathol Microbiol 36: 45–47 (1993 | Other underlying disease: Sclerema neonatorum |
| 245 | Del Pozzo-Magaña BR, Ho N: Subcutaneous Fat Necrosis of the Newborn: A 20-Year Retrospective Study. Pediatr Dermatol 33: e353-e355 (2016) | Retrospective case series without precise information on individual cases |
| 246 | Della Corte G, Sbano E, Galasso F, Altamura V: Adiponecrosi sottocutanea del neonato. G Ital Dermatol Venereol 125: 389–392 (1990) | No access to the full text possible |
| 247 | Dieckhoff J, Hempel HC, Koch R: The traumatic subcutaneous fat necrosis in the new-born infant. Kinderarztl Prax 27: 443–452 (1959) | No case report |
| 248 | Dudink J, Roeten BM, van der Meer-Kappelle LH, Walther FJ: Pijnlijke huidafwijkingen bij twee pasgeborenen: neonatale  subcutane vetnecrose. Ned Tijdschr Geneeskd 147: 2337–2340 (2003) | Double case report |
| 249 | Farooque A, Moss C, Zehnder D, Hewison M, Shaw NJ: Expression of 25-hydroxyvitamin D3-1alpha-hydroxylase in  subcutaneous fat necrosis. Br J Dermatol 160: 423–425 (2009) | No case report |
| 250 | Favus MJ: Primer on the metabolic bone diseases and disorders of mineral metabolism: Miscellaneous causes of  hypercalcemia. Lippincott-Raven, Philadelphia, S.477 (1996) | Other underlying disease: metabolic bone disease |
| 251 | Feng Z, Guo B, Zhang Z: Subcutaneous fat necrosis of the newborn associated with hypercalcemia after therapeutic  hypothermia. J La State Med Soc 166: 97–99 (2014) | No case report |
| 252 | Ferlazzo A: Considerazioni su un caso di adiponecrosi del sottocutaneo in postmaturo. Rass Clin Sci 40: 104–108 (1964) | No access to the full text possible |
| 253 | Feyling T: Subkutan fettvevsnekrose (adiponecrosis subcutanea) med hypercalcemi hos nyfødte. Tidsskr Nor  Laegeforen 90: 1077–1080 (1970) | Other language except English, French, Spanish, Italian. No Abstract available in English |
| 254 | Filippi L, Catarzi S, Padrini L, Fiorini P, La Marca G, Guerrini R, Donzelli GP: Strategies for reducing the incidence of skin  complications in newborns treated with whole-body hypothermia. J Matern Fetal Neonatal Med 25: 2115–2121 (2012) | No case report |
| 255 | Fliers EA, Sillevis Smitt JH, van Zaane DJ: Adiponecrosis subcutanea neonatorum met hypercalciëmie. Ned Tijdschr  Geneeskd 128: 401–404 (1984) | Other language except English, French, Spanish, Italian. No Abstract available in English |
| 256 | Flood JM, Beard WJ: Subcutaneous fat necrosis of the new-born; a case report. Guthrie Clin Bull 17: 132–134 (1948) | No access to the full text possible |
| 257 | Flory CM: Fat necrosis of the newborn; report of a case with necrosis of the subcutaneous and visceral fat. Arch Pathol  (Chic.) 45: 278–288 (1948) | No access to the full text possible |
| 258 | Freeman NV: Surgery of the newborn. Churchill Livingstone, Edinburgh, S.824 (1994) | No case report |
| 259 | Fretzin DF, Arias AM: Sclerema neonatorum and subcutaneous fat necrosis of the newborn. Pediatr Dermatol 4: 112–  122 (1987) | No case report |
| 260 | Frumkin A, Mogilner B: Subcutaneous fat necrosis of the newborn. Harefuah 118: 117–118 (1990) | No case report |
| 261 | Gold DL: Visual diagnosis: perceived fevers and back pain in a 1-week-old Infant. Pediatr Rev 32: 27–30 (2011) | No inclusion criteria met |
| 262 | Golden ET, Dickson P, Simoneaux S: Brown fat necrosis with calcifications in the newborn: Risk factors, radiographic findings, and clinical course. Indian J Radiol Imaging 28: 107–110 (2018) | Case series without precise information on individual cases |
| 263 | Grassi S, Borroni RG, Brazzelli V: Panniculitis in children. G Ital Dermatol Venereol 148: 371–385 (2013) | No case report |
| 264 | Grewal SK, Elston DM: https://​emedicine.medscape.com​/​article/​1081910-overview (21.05.2018) | No case report |
| 265 | Gualandri L, Mapelli ET, Danese P, Menni S: Subcutaneous fat necrosis in a newborn. G Ital Dermatol Venereol 150:  766–768 (2015) | No access to the full text possible |
| 266 | Gupta RK, Naran S, Selby RE: Fine needle aspiration cytodiagnosis of subcutaneous fat necrosis of newborn. A case  report. Acta Cytol 39: 759–761 (1995) | No access to the full text possible |
| 267 | Haan TR de, Grooters E, Frijns JHM, Walther FJ: Unilateral submandibular suppurative sialadenitis in a premature infant.  Acta Paediatr 92: 1491–1493 (2003) | Other underlying disease: sialadenitis |
| 268 | Hakan N, Aydin M, Zenciroglu A: Reply to the correspondence letter by Dr. Mitra S. to "alendronate for the treatment of  hypercalcaemia due to neonatal subcutaneous fat necrosis". Eur J Pediatr 170: 1357–1358 (2011) | No case report |
| 269 | Hallez GL: Nouvelle contribution à l'étude de la stéatonecrose souscutanée traumatique des nouveau-nés; anatomie  pathologique et pathogénie. Nourrisson 38: 287–296 (1950) | No case report |
| 270 | Hallman N, Hjelt L, Backman A: Neonatal subcutaneous fat necrosis in children of diabetic mothers. Annales paediatriae  Fenniae 2: 19–30 (1956) | No abstract or full text available |
| 271 | Hansen KN, Balslev T, Lyngbye TJ, Olesen B, Ebbesen F: Subkutan fedtnekrose med hyperkalcaemi. Ugeskr Laeger  157: 5398–5399 (1995) | No access to the full text possible |
| 272 | Hasan R: Hypercalcemia after subcutaneous fat necrosis in a newborn. J Investig Med 61: 398 (2013) | No case report |
| 273 | He Y, Perry B, Bi M, Sun H, Zhao T, Li Y, Sun C: Allosteric regulation of the calcium-sensing receptor in obese  individuals. Int J Mol Med 32: 511–518 (2013) | No case report |
| 274 | Hornick JL, Fletcher CDM: Intraabdominal cystic lymphangiomas obscured by marked superimposed reactive changes:  clinicopathological analysis of a series. Hum Pathol 36: 426–432 (2005) | Other underlying disease: cystic lymphangioma |
| 275 | Hsieh WS, Yang PH, Chao HC, Lai JY: Neonatal necrotizing fasciitis: a report of three cases and review of the literature.  Pediatrics 103: 1-6 (1999) | Other underlying disease: necrotizing fasciitis |
| 276 | Ichimiya H, Arakawa S, Sato T, Shimada T, Chiba M, Soma Y, Mizoguchi M, Tomonari K, Iwasaka H, Hatano Y,  Okamoto O, Fujiwara S: Involvement of brown adipose tissue in subcutaneous fat necrosis of the newborn. Dermatology  223: 207–210 (2011) | No case report |
| 277 | Jacobs, Susan E. Berg, Marie. Hunt, Rod. TarnowMordi, William O. Inder, Terrie E. Davis, Peter G.: Cooling for  newborns with hypoxic ischaemic encephalopathy. EBM Reviews - Cochrane Database of Systemic Reviews 3 (2013) | No case report |
| 278 | Jong AL de, Ravel E, Forte V, Taylor G: Head and neck manifestations of infantile subcutaneous fat necrosis.  J Otolaryngol 31: 257–260 (2002) | No abstract or full text available |
| 279 | Kattan H, Sakati N, Abduljabbar J, Al-Eisa A, Nou-Nou ML: Subcutaneous Fat Necrosis as an Unusual Presentation of  Child Abuse. Ann Saudi Med 15: 162–164 (1995) | Not subcutaneous adipose tissue necrosis but child abuse |
| 280 | Kenani N, Mebaza A, Denguezli M, Sriha B, Belajouza C, Nouira R: Subcutaneous fat necrosis of the newborn. Eur  J Dermatol 17: 99–100 (2007) | No abstract or full text available |
| 281 | Kesan K, Kothari P, Gupta R, Gupta A, Karkera P, Ranjan R, Mutkhedkar K, Sandlas G: Closure of large  meningomyelocele wound defects with subcutaneous based pedicle flap with bilateral V-Y advancement: our experience  and review of literature. Eur J Pediatr Surg 25: 189–194 (2015) | Other underlying disease: meningomyelocele |
| 282 | Koshizuka K, Koike M, Kubota T, Said J, Binderup L, Koeffler HP: Novel vitamin D3 analog (CB1093) when combined  with paclitaxel and cisplatin inhibit growth of MCF-7 human breast cancer cells in vivo. Int J Oncol 13: 421–428 (1998) | Other underlying disease: breast cancer |
| 283 | Kosloske AM, Bartow SA: Debridement of periumbilical necrotizing fasciitis: importance of excision of the umbilical  vessels and urachal remnant. J Pediatr Surg 26: 808–810 (1991) | Other underlying disease: necrotizing fasciitis |
| 284 | Kural N., Tel N.: Subcutaneous fat necrosis of the newborn. Cocuk Sagligi ve Hastaliklari Dergisi 29: 71–74 (1986) | No abstract or full text available |
| 285 | Levene MI, Wigglesworth JS, Desai R: Pulmonary fat accumulation after intralipid infusion in the preterm infant. Lancet 2:  815–818 (1980) | Other underlying disease: pulmonary fat accumulation |
| 286 | Liang WH, Lin JT, Hsiao LC, Lin ST: Congenital muscular dystrophy: report of one case. Zhonghua Min Guo Xiao Er Ke  Yi Xue Hui Za Zhi 36: 442–444 (1995) | Other underlying disease: congenital muscular dystrophy |
| 287 | Lin EP, Aronson LA: Successful resuscitation of bupivacaine-induced cardiotoxicity in a neonate. Paediatr Anaesth 20:  955–957 (2010) | Other underlying disease: bupivacaine-induced cardiotoxicity |
| 288 | Lindlar F, Misgeld V: Die Adiponecrosis subcutanea neonatorum unter lipoidchemischem Aspekt. Hautarzt 18: 115–118  (1967) | No case report |
| 289 | Lipets ME, Shibaeva LN, Prokof'eva NM, Gavrikov VV: Nekroz podkozhnoĭ kletchatki novorozhdennykh. Vestn Dermatol  Venerol 8: 56–59 (1985) | Other language except English, French, Spanish, Italian. No Abstract available in English |
| 290 | Liu DC, Wernikoff S: Red nodules in an infant. Subcutaneous fat necrosis. Arch Dermatol 122: 822-826 (1986) | No abstract or full text available |
| 291 | Liu FT, Dobry MM, Shames BS, Goltz RW: Subcutaneous nodules and hypercalcemia in an infant. Subcutaneous fat  necrosis of the newborn. Arch Dermatol 129: 898-901-2 (1993) | No abstract or full text available |
| 292 | Llamas-Velasco M, Requena L: Panniculitis with crystals induced by etanercept subcutaneous injection. J Cutan Pathol  42: 413–415 (2015) | Other underlying disease |
| 293 | Locham KK, Parmar GS: Subcutaneous fat necrosis of newborn. Indian Pediatr 37: 102 (2000) | No abstract or full text available |
| 294 | Lombet J: Commentary on 'Pamidronate: treatment for severe hypercalcemia in neonatal subcutaneous fat necrosis' by  Alos N. et al., Hormone Research 2006;65:289-294. Horm Res Paediatr 70: 254-5; author reply 256 (2008) | No case report |
| 295 | Longo MI, Hernanz JM, Lecona M, Lázaro P: Off-center fold: dark-red plaques on the scalp of a newborn. Arch Dermatol  136: 1559–1564 (2000) | No inclusion criteria met |
| 296 | López V, Alonso V, Rayón JM, Monteagudo C, Jordá E: Usefulness of fine-needle aspiration in subcutaneous fat  necrosis of the newborn diagnosis. Pediatr Dermatol 27: 317–318 (2010) | No case report |
| 297 | DeLuca R, Reverdin A, Tissot C[A], Pfister RE[A]: Joint Annual Meeting of the Swiss-Society-for-Pediatrics.  Swiss Med Wkly 140: 21–22 (2010) | No inclusion criteria met |
| 298 | Lund JJ, Xia L, Kerr S, Stratman EJ, Patten SF: The utility of a touch preparation in the diagnosis of fluctuant  subcutaneous fat necrosis of the newborn. Pediatr Dermatol 26: 241–243 (2009) | No case report |
| 299 | Mahé E, Prost Y de: La cytostéatonécrose du nouveau-né. Ann Dermatol Venereol 134: 494-499 (2007) | No access to the full text possible |
| 300 | Mahé E, Descamps JV, Belaïch S, Crickx B: La cytostéatonécrose du nouveau-né. Presse Med 31: 612–616 (2002) | No case report |
| 301 | Marani M, Biasini A, Lotti V, Ciotti F, Miano A, Montaguti A, Ponti R: Adiponecrosi del neonato associata a ipercalcemia  e dislipidemia (descrizione di un caso). Pediatr Med Chir 4: 563–564 (1982) | No access to the full text possible |
| 302 | Marconi V, Mofid MZ, McCall C, Eckman I, Nousari HC: Primary hyperoxaluria: report of a patient with livedo reticularis and digital infarcts. J Am Acad Dermatol 46: 16-18 (2002) | Other underlying disease: primary hyperoxaluire |
| 303 | Masi A BL: L'adiponecrosi del tessuto sottocutaneo del neonato; contributo statistico e clinico. Riv Clin Pediatr 57: 362–  416 (1956) | No access to the full text possible |
| 304 | McDonald R: Subcutaneous fat necrosis and sclerema neonatorum. S Afr Med J 29: 1007–1012 (1955) | No abstract or full text available |
| 305 | McNeilly JD, Boal R, Shaikh MG, Ahmed SF: Frequency and aetiology of hypercalcaemia. Arch Dis Child 101: 344–347  (2016) | No case report |
| 306 | Metz SA, Hassal E: PGE, hypercalcemia, and subcutaneous fat necrosis. J Pediatr 97: 336 (1980) | No case report |
| 307 | Michael AF, Hong R, West CD: Hypercalcemia in infancy associated with subcutaneous fat necrosis and calcification.  Am J Dis Child 104: 235–244 (1962) | No abstract or full text available |
| 308 | Milanesi E, Loda C, Drera B, Poggiani C: Hypercalcemia and nephrocalcinosis complicating subcutaneous fat necrosis in  a newborn after therapeutic hypothermia. Minerva Pediatr 68: 316–317 (2016) | No access to the full text possible |
| 309 | Montes LF: Subcutaneous fat necrosis of the newborn with eosinophilic granules. J Cutan Pathol 35: 699-700 (2008) | No case report |
| 310 | Morris RB: Subcutaneous fat necrosis of the new-born. Med J Aust 2: 696-698 (1960) | No abstract or full text available |
| 311 | Hamodat M: http://​www.pathologyoutlines.com​/​topic/​skinnontumorsubcutaneousfatnecrosisofnewborn.html (21.05.2018) | Double case report |
| 312 | Muscardin LM, Guadagni AM, Conti G, De Pietro U: Subcutaneous adiponecrosis in the newborn etiopathogenetic  consideration on a clinical case. Chronica Dermatologica 17: 347–354 (1986) | No access to the full text possible |
| 313 | Nseir V, Bradauskaite G, Pedroza M, Minimo C, Zaki R, Chewaproug D: A Rare Case of Calciphylaxis in an Orthotopic  Liver Transplant Recipient with Acute Kidney Injury. Exp Clin Transplant: 1–4 (2018) | Other underlying disease: calciphylaxis |
| 314 | Oehlert W, Wecke H, Sütterle H: Die Fettgewebsnekrose des Neugeborenen bei Diabetes mellitus der Mutter. Zentralbl  Allg Pathol 107: 499–505 (1965) | No abstract or full text available |
| 315 | Padiatelles K, Konstantopoulus K, Tsitsilianos D: Idiomorphos periptosis ektetamenes nekroseos hypoderieu lipous.  Hell Iatr 33: 455–462 (1964) | Other language except English, French, Spanish, Italian. No Abstract available in English |
| 316 | Palomo Arellano A, Alvarez Cárdenas MC, González Eusebio B, Gil Pascual B: Nódulos subcutáneos en un recién  nacido. An Esp Pediatr 48: 539–540 (1998) | No access to the full text possible |
| 317 | Park SH, Kim S-C: Sclerema Neonatorum in a Full-Term Infant Showing Favorable Prognosis. Ann Dermatol 29: 790–  793 (2017) | Other underlying disease: Sclerema neonatorum |
| 318 | Paschoal LH, Dall'Aglio FF, Cohen S, Freitas JP de, Saba LB: Adiponecrose do recém-nascido: apresentação de dois  casos. Rev Paul Med 97: 80–83 (1981) | No access to the full text possible |
| 319 | Passaro G: Contributo allo studio della steatonecrosi del sottocutaneo nel neonato. Arch Ital Pediatr Pueric 17: 433–451  (1955) | No abstract or full text available |
| 320 | Pasyk K: Martwica tkanki tłuszczowej podskórnej noworodków (badania w mikroskopie świetlnym i elektronowym). Folia  Med Cracov 20: 327–344 (1978) | Other language except English, French, Spanish, Italian. No Abstract available in English |
| 321 | Pasykowa K, Jelonek A, Depowski M: Adiponecrosis subcutanea neonatorum. Przegl Lek 30: 392–394 (1973) | Other language except English, French, Spanish, Italian. No Abstractavailable in English |
| 322 | Perme T, Mali S, Vidmar I, Gvardijančič D, Blumauer R, Mishaly D, Grabnar I, Nemec G, Grosek S: Prolonged  prostaglandin E1 therapy in a neonate with pulmonary atresia and ventricular septal defect and the development of antral  foveolar hyperplasia and hypertrophic pyloric stenosis. Ups J Med Sci 118: 138–142 (2013) | Other underlying disease: pulmonary atresia, ventricular septal defect, foveolar hyperplasia, and hypertrophic pyloric stenosis. |
| 323 | Pettinato G, Manivel JC, Wick MR, Dehner LP: Classical and cellular (atypical) congenital mesoblastic nephroma: a  clinicopathologic, ultrastructural, immunohistochemical, and flow cytometric study. Hum Pathol 20: 682–690 (1989) | Other underlying disease: mesoblastic nephroma |
| 324 | Phelan MS, Lams P: Massive pulmonary calcification in two infants with congenital cardiac lesions. Clin Radiol 34: 381–  384 (1983) | Other underlying disease: pulmonary calcifications in cardiac disease. |
| 325 | Pitchumoni CS, Agarwal N, Jain NK: Systemic complications of acute pancreatitis. Am J Gastroeneterol 83: 597–606  (1988) | Other underlying disease: Acute pancreatitis |
| 326 | Polcari IC, Stein SL: Panniculitis in childhood. Dermatol Ther 23: 356–367 (2010) | No inclusion criteria met |
| 327 | Possega R, Massaro A, Baciocco C, Napoli G, Bettoschi U, Carli C: L'adiponecrosi sottocutanea del neonato.  Descrizione di un caso clinico. Pathologica 72: 573–581 (1980) | No access to the full text possible |
| 328 | Proks C: Fettkristalle im Unterhautfettgewebe bei Neugeborenen, Sclerema neonatorum und Adiponecrosis cutis neonatorum. Virchows Arch A Pathol Anat Histol 337: 584–592 (1964) | No abstract or full text available |
| 329 | Puzenat E, Aubin F, Zyka F, Fromentin C, Humbert P: Cytostéatonécrose du nouveau-né compliquée d'une  hypercalcémie. Ann Dermatol Venereol 127: 1087–1089 (2000) | No access to the full text possible |
| 330 | Quesada-Cortés A, Campos-Muñoz L, Díaz-Díaz RM, Casado-Jiménez M: Cold panniculitis. Dermatol Clin 26: 485-489  (2008) | Other underlying disease: cold panniculitis |
| 331 | Ramsay TG, Stoll MJ, Conde-Aguilera JA, Caperna TJ: Peripheral tumor necrosis factor α regulation of adipose tissue  metabolism and adipokine gene expression in neonatal pigs. Vet Res Commun 37: 1–10 (2013) | Animal study |
| 332 | Requena L, Sánchez Yus E: Panniculitis. Part II. Mostly lobular panniculitis. J Am Acad Dermatol 45: 325-364 (2001) | No inclusion criteria met |
| 333 | Requena L: Normal subcutaneous fat, necrosis of adipocytes and classification of the panniculitides. Semin Cutan Med  Surg 26: 66–70 (2007) | No case report |
| 334 | Ricardo-Gonzalez RR, Lin JR, Mathes EF, McCalmont TH, Pincus LB: Neutrophil-rich subcutaneous fat necrosis of the  newborn: A potential mimic of infection. J Am Acad Dermatol 75: 177-185 (2016) | Case series without precise information on individual cases |
| 335 | Rommani SR, M'farrej MK, Nechi S, Kourda M, Fazaa B, Zermani R: Lésion sous-cutanée du nouveau-né. Ann Pathol  33: 360–362 (2013) | No access to the full text possible |
| 336 | Salas Valién JS, Ribas Ariño MT, Egido Romo M, Palau Benavides MT: Subcutaneous fat necrosis of newborn children.  Histol Histopathol 5: 1–5 (1990) | No access to the full text possible |
| 337 | Sato T, Takahashi K, Kojima M: Sclerema neonatorum associated with systemic fibrosis and endocardial fibroelastosis.  Acta Pathol Jpn 27: 917–925 (1977) | Other underlying disease: systemic fibrosis in sclerema neonatorum. |
| 338 | Schenk JP, Schrader C, Furtwängler R, Ko HS, Leuschner I, Graf N, Troeger J: MRT-Morphologie und Staging des  kongenitalen mesoblastischen Nephroms: Auswertung einer Fallsammlung mit 20 Patienten. Rofo 177: 1373–1379  (2005) | Other underlying disease: congenital mesoblastic nephroma. |
| 339 | Schulzke S, Büchner S, Fahnenstich H: Subcutaneous fat necrosis of the newborn. Swiss Med Wkly 135: 122–123  (2005) | No abstract or full text available |
| 340 | Setchell KDR, Heubi JE, Bove KE, O'Connell NC, Brewsaugh T, Steinberg SJ, Moser A, Squires RH: Liver disease  caused by failure to racemize trihydroxycholestanoic acid: gene mutation and effect of bile acid therapy.  Gastroenterology 124: 217–232 (2003) | Other underlying disease: liver disease |
| 341 | Shackelford GD, Barton LL, McAlister WH: Calcified subcutaneous fat necrosis in infancy. J Can Assoc Radiol 26: 203–  207 (1975) | No abstract or full text available |
| 342 | Shek YH, Tucker MC, Viciana AL, Manz HJ, Connor DH: Malassezia furfur--disseminated infection in premature infants.  Am J Clin Pathol 92: 595–603 (1989) | Other underlying disease: Malassezia furfur |
| 343 | Shinskiĭ GE, Timofeeva ED: Podkozhnozhirovoĭ nekroz novorozhdennykh. Vestn Dermatol Venerol 42: 76–80 (1968) | Other language except English, French, Spanish, Italian. No Abstract available in English |
| 344 | Shivaprasad HL: Hepatitis associated with Clostridium difficile in an ostrich chick. Avian Pathol 32: 57–62 (2003) | Other underlying disease: hepatitis and Clostridium difficile |
| 345 | Singh A, Sharma S, Vermani S, Khunger N: Subcutaneous fat necrosis of the new born. Indian J Pathol Microbiol 50:  849–850 (2007) | No abstract or full text available |
| 346 | So WC, Lin CH, Lee JY: Subcutaneous fat necrosis of newborn: report of two cases. Zhonghua Min Guo Xiao Er Ke Yi  Xue Hui Za Zhi 32: 239–243 (1991) | No access to the full text possible |
| 347 | Souissi R, Kourda M, Rouatbi M: Cas clinique: cytostéatonécrose néo-natale. A propos d'un cas. Tunis Med 68: 693–696  (1990) | No access to the full text possible |
| 348 | Spanakis EK, Sellmeyer DE: Nonuremic calciphylaxis precipitated by teriparatide rhPTH(1-34) therapy in the setting of  chronic warfarin and glucocorticoid treatment. Osteoporos Int 25: 1411–1414 (2014) | Other underlying disease: calciphylaxis |
| 349 | Spinelli G, Vercellotti E: L'adiponecrosi sottocutanea del neonato. Presentazione di un caso. Minerva Med 70: 2175–  2177 (1979) | No access to the full text possible |
| 350 | Steiness I: Subcutaneous fat necrosis in the newborn and maternal diabetes mellitus. Ugeskr Laeger 124: 368 (1962) | Other language except English, French, Spanish, Italian. No Abstract available in English |
| 351 | Steiness I: Subcutaneous fat necrosis of the newborn (adiponecrosis subcutanea neonatorum) and maternal diabetes  mellitus. Acta Med Scand 170: 411–416 (1961) | No abstract or full text available |
| 352 | Strohm B, Hobson A, Brocklehurst P, Edwards AD, Azzopardi D: Subcutaneous fat necrosis after moderate therapeutic  hypothermia in neonates. Pediatrics 128: e450-e452 (2011) | Case series without precise information on individual cases |
| 353 | Suprun H, Freundlich E: Fatal familial steatosis of myocardium, liver and kidneys in three siblings. Acta Paediatr Scand  70: 247–252 (1981) | Other underlying disease: familial myocardial steatosis |
| 354 | Sydow G von: Calmettevaccination med anmärkningsvärt förlopp hos nyfödd med subkutana fettnekroser. Nord Med 44:  1324–1325 (1950) | Other language except English, French, Spanish, Italian. No Abstract available in English |
| 355 | Taieb A, Douard D, Sarlangue J, Fontan I, Nelson JR, Martin C, Maleville J: Trois cas de cytostéatonécrose néo-natale.  Discussion physiopathologique. Presse Med 15: 2197–2200 (1986) | No access to the full text possible |
| 356 | Taïeb A, Ball M: Quel est votre diagnostic? Cytosteatonecrosis neonatale. Ann Dermatol Venereol 116: 47–49 (1989) | No case report |
| 357 | Taïeb A, Douard D, Maleville J: Subcutaneous fat necrosis and brown fat deficiency. J Am Acad Dermatol 16: 624–625  (1987) | No exact case details |
| 358 | Tjoeng H, Najaf T: Index of Suspicion in the Nursery. NeoReviews 9: e590-e593 (2008) | No inclusion criteria met |
| 359 | Treat J: https://​www.dermatologyadvisor.com​/​dermatology/​subcutaneous-fat-necrosis-of-the-newborn/​article/​691472/​  (21.05.2018) | No case report |
| 360 | Tseng M-H, Chu S-M, Cheng C-J, Lien R, Shih I-S, Lin S-H: An infant with multiple subcutaneous nodules,  hypercalcemia, and nephrocalcinosis. Pediatr Nephrol 28: 2283–2287 (2013) | No case report |
| 361 | Turba F, Bianchi C, Cella D, Rondanini GF: Trombocitosi e adiponecrosi sottocutanea neonatale. Minerva Pediatr 46:  343–346 (1994) | No access to the full text possible |
| 362 | Urban J, Janniger CK, Toruniowa B, Suszka-Cichosz M: Subcutaneous fat necrosis of the newborn. Cutis 54: 383–385  (1994) | No abstract or full text available |
| 363 | Varastehpour A, Radaelli T, Minium J, Ortega H, Herrera E, Catalano P, Hauguel-de Mouzon S: Activation of  phospholipase A2 is associated with generation of placental lipid signals and fetal obesity. J Clin Endocrinol Metab 91:  248–255 (2006) | No inclusion criteria met |
| 364 | Velter C, Lipsker D: Panniculites cutanées. Rev Med Interne 37: 743–750 (2016) | No case report |
| 365 | Vera LA, Zaeri N, Hurt H: Picture of the month. Subcutaneous fat necrosis of the newborn. Am J Dis Child 145: 1047–  1048 (1991) | No case report |
| 366 | Vonk J, Janssens PM, Demacker PN, Folkers E: Subcutaneous fat necrosis in a neonate, in association with aberrant plasma lipid and lipoprotein values. J Pediatr 123: 462–464 (1993) | Double case report |
| 367 | Wang H, Zhang Q, Chai Y, Liu Y, Li F, Wang B, Zhu C, Cui J, Qu H, Zhu M: 1,25(OH)2D3 downregulates the Toll-like  receptor 4-mediated inflammatory pathway and ameliorates liver injury in diabetic rats. J Endocrinol Invest 38: 1083–  1091 (2015) | No inclusion criteria met |
| 368 | Washington K, Gossage DL, Gottfried MR: Pathology of the pancreas in severe combined immunodeficiency and  DiGeorge syndrome: acute graft-versus-host disease and unusual viral infections. Hum Pathol 25: 908–914 (1994) | Other underlying disease: DiGeorge syndrome |
| 379 | Wehinger H: Spätrachitis nach Vitamin D-Uberempfindlichkeit bei Adiponekrosis subcutanea in der  Neugeborenenperiode. Z Kinderheilkd 107: 42–52 (1969) | No abstract or full text available |
| 370 | Weigel W, Hayek WH, Bens G: Osteoarthritis bei Neugeborenen. Radiologische Diagnostik und Verlaufsbeobachtungen.  Rofo 130: 68–76 (1979) | Other underlying disease: osteoarthritis |
| 371 | Welsh KM, Smoller BR, Holbrook KA, Johnston K: Restrictive dermopathy. Report of two affected siblings and a review  of the literature. Arch Dermatol 128: 228–231 (1992) | Other underlying disease: restrictive dermopathy |
| 372 | Wessling-Assmann K, Traupe H, Bonsmann G, Metze D: Subkutane Fettgewebsnekrose des Neugeborenen. J Dtsch  Dermatol Ges 1: 297–299 (2003) | No case report |
| 373 | Wick MR: Panniculitis: A summary. Semin Diagn Pathol 34: 261–272 (2017) | No inclusion criteria met |
| 374 | Wilmer WA, Magro CM: Calciphylaxis: emerging concepts in prevention, diagnosis, and treatment. Semin Dial 15: 172–  186 (2002) | Other underlying disease: calciphylaxis |
| 375 | Yajnik CS: The lifecycle effects of nutrition and body size on adult adiposity, diabetes and cardiovascular disease. Obes  Rev 3: 217–224 (2002) | Age of the child >2 years |
| 376 | Yılmaz R, Kundak AA, Sezer T, Özer S, Esmeray H, Kazancı NÖ: Idiopathic infantile hypercalcemia or an extrapulmonary complication of tuberculosis? Tuberk Toraks 61: 43–46 (2013) | Other underlying disease: tuberculosis |
| 377 | Yimesel M, Assefa G: Extensive subcutaneous soft tissue calcification in a neonate following hypothermia: case report.  East Afr Med J 77: 231–232 (2000) | No access to the full text possible |
| 378 | Zeb A, Darmstadt GL: Sclerema neonatorum: a review of nomenclature, clinical presentation, histological features,  differential diagnoses and management. J Perinatol 28: 453–460 (2008) | Other underlying disease: Sclerema neonatorum |
| 379 | Zhou W, Wiesenthal A, Carr V, Allison A, Kelly B, Gibson B: A firm plaque on the back of a newborn. Dermatol Online J  16: 11 (2010) | No access to the full text possible |
| 380 | Zviagintseva SG, Doletskii SI, Vikhireva ZN, Ostromoukhova GA, Gromova RV: On so-called subcutaneous fat necrosis in newborn infants. Pediatriia 42: 68–73 (1963) | Other language except English, French, Spanish, Italian. No Abstract available in English |
